# Antibody and T-Cell Response to Bivalent Booster SARS-CoV-2 Vaccines in People With Compromised Immune Function: COVERALL-3 Study

**DOI:** 10.1093/infdis/jiae291

**Published:** 2024-06-07

**Authors:** Alain Amstutz, Frédérique Chammartin, Annette Audigé, Anna L Eichenberger, Dominique L Braun, Patrizia Amico, Marcel P Stoeckle, Barbara Hasse, Matthaios Papadimitriou-Olivgeris, Oriol Manuel, Cédric Bongard, Macé M Schuurmans, René Hage, Dominik Damm, Michael Tamm, Nicolas J Mueller, Andri Rauch, Huldrych F Günthard, Michael T Koller, Christof M Schönenberger, Alexandra Griessbach, Niklaus D Labhardt, Roger D Kouyos, Alexandra Trkola, Katharina Kusejko, Heiner C Bucher, Irene A Abela, Matthias Briel, Benjamin Speich, Swiss HIV Cohort Study, I Abela, K Aebi-Popp, A Anagnostopoulos, M Battegay, E Bernasconi, D L Braun, H C Bucher, A Calmy, M Cavassini, A Ciuffi, G Dollenmaier, M Egger, L Elzi, J Fehr, J Fellay, H Furrer, C A Fux, H F Günthard, A Hachfeld, D Haerry, B Hasse, H H Hirsch, M Hoffmann, I Hösli, M Huber, D Jackson-Perry, C R Kahlert, L Kaiser, O Keiser, T Klimkait, R D Kouyos, H Kovari, K Kusejko, N Labhardt, K Leuzinger, B Martinez de Tejada, C Marzolini, K J Metzner, N Müller, J Nemeth, D Nicca, J Notter, P Paioni, G Pantaleo, M Perreau, A Rauch, L Salazar-Vizcaya, P Schmid, R Speck, M Stöckle, P Tarr, A Trkola, G Wandeler, M Weisser, S Yerly, the Swiss Transplant Cohort, Patrizia Amico, John-David Aubert, Vanessa Banz, Sonja Beckmann, Guido Beldi, Christoph Berger, Ekaterine Berishvili, Annalisa Berzigotti, Isabelle Binet, Pierre-Yves Bochud, Sanda Branca, Heiner C Bucher, Emmanuelle Catana, Anne Cairoli, Yves Chalandon, Sabina De Geest, Olivier De Rougemont, Sophie De Seigneux, Michael Dickenmann, Joëlle Lynn Dreifuss, Michel Duchosal, Thomas Fehr, Sylvie Ferrari-Lacraz, Christian Garzoni, Déla Golshayan, Nicolas Goossens, Fadi Haidar, Jörg Halter, Dominik Heim, Christoph Hess, Sven Hillinger, Hans H Hirsch, Patricia Hirt, Linard Hoessly, Günther Hofbauer, Uyen Huynh-Do, Franz Immer, Michael Koller, Bettina Laesser, Frédéric Lamoth, Roger Lehmann, Alexander Leichtle, Oriol Manuel, Hans-Peter Marti, Michele Martinelli, Valérie McLin, Katell Mellac, Aurélia Merçay, Karin Mettler, Nicolas J Mueller, Ulrike Müller-Arndt, Beat Müllhaupt, Mirjam Nägeli, Graziano Oldani, Manuel Pascual, Jakob Passweg, Rosemarie Pazeller, Klara Posfay-Barbe, Juliane Rick, Anne Rosselet, Simona Rossi, Silvia Rothlin, Frank Ruschitzka, Thomas Schachtner, Stefan Schaub, Alexandra Scherrer, Aurelia Schnyder, Macé Schuurmans, Simon Schwab, Thierry Sengstag, Federico Simonetta, Susanne Stampf, Jürg Steiger, Guido Stirnimann, Ueli Stürzinger, Christian Van Delden, Jean-Pierre Venetz, Jean Villard, Julien Vionnet, Madeleine Wick, Markus Wilhelm, Patrick Yerly

**Affiliations:** Division of Clinical Epidemiology, Department of Clinical Research, University Hospital Basel, University of Basel, Basel, Switzerland; Oslo Center for Biostatistics and Epidemiology, Oslo University Hospital, University of Oslo, Oslo, Norway; Population Health Sciences, Bristol Medical School, University of Bristol, Bristol, United Kingdom; Division of Clinical Epidemiology, Department of Clinical Research, University Hospital Basel, University of Basel, Basel, Switzerland; Institute of Medical Virology, University of Zurich, Zurich, Switzerland; Department of Infectious Diseases, Inselspital, Bern University Hospital, University of Bern, Bern, Switzerland; Institute of Medical Virology, University of Zurich, Zurich, Switzerland; Department of Infectious Diseases and Hospital Epidemiology, University Hospital Zurich, Zurich, Switzerland; Clinic for Transplantation Immunology and Nephrology, University Hospital Basel, Basel, Switzerland; Division of Infectious Diseases and Hospital Epidemiology, University Hospital Basel, University of Basel, Basel, Switzerland; Department of Infectious Diseases and Hospital Epidemiology, University Hospital Zurich, Zurich, Switzerland; Infectious Diseases Service and Transplantation Center, Lausanne University Hospital, Lausanne, Switzerland; Infectious Diseases Service and Transplantation Center, Lausanne University Hospital, Lausanne, Switzerland; Division of Pulmonology, Department of Medicine, University Hospital of Lausanne, Lausanne, Switzerland; Division of Pulmonology, University Hospital Zurich, Zurich, Switzerland; Division of Pulmonology, University Hospital Zurich, Zurich, Switzerland; Division of Pulmonology, University Hospital Zurich, Zurich, Switzerland; Clinic of Respiratory Medicine and Pulmonary Cell Research, University Hospital Basel, Basel, Switzerland; Department of Infectious Diseases and Hospital Epidemiology, University Hospital Zurich, Zurich, Switzerland; Department of Infectious Diseases, Inselspital, Bern University Hospital, University of Bern, Bern, Switzerland; Institute of Medical Virology, University of Zurich, Zurich, Switzerland; Department of Infectious Diseases and Hospital Epidemiology, University Hospital Zurich, Zurich, Switzerland; Clinic for Transplantation Immunology and Nephrology, University Hospital Basel, Basel, Switzerland; Swiss Transplant Cohort Study, University Hospital Basel, Basel, Switzerland; Division of Clinical Epidemiology, Department of Clinical Research, University Hospital Basel, University of Basel, Basel, Switzerland; Division of Clinical Epidemiology, Department of Clinical Research, University Hospital Basel, University of Basel, Basel, Switzerland; Division of Clinical Epidemiology, Department of Clinical Research, University Hospital Basel, University of Basel, Basel, Switzerland; Institute of Medical Virology, University of Zurich, Zurich, Switzerland; Department of Infectious Diseases and Hospital Epidemiology, University Hospital Zurich, Zurich, Switzerland; Institute of Medical Virology, University of Zurich, Zurich, Switzerland; Department of Infectious Diseases and Hospital Epidemiology, University Hospital Zurich, Zurich, Switzerland; Division of Clinical Epidemiology, Department of Clinical Research, University Hospital Basel, University of Basel, Basel, Switzerland; Institute of Medical Virology, University of Zurich, Zurich, Switzerland; Department of Infectious Diseases and Hospital Epidemiology, University Hospital Zurich, Zurich, Switzerland; Division of Clinical Epidemiology, Department of Clinical Research, University Hospital Basel, University of Basel, Basel, Switzerland; Department of Health Research Methods, Evidence, and Impact, McMaster University, Hamilton, Ontario, Canada; Division of Clinical Epidemiology, Department of Clinical Research, University Hospital Basel, University of Basel, Basel, Switzerland

**Keywords:** SARS-CoV-2, HIV, organ transplant, vaccine, bivalent vaccine

## Abstract

**Background:**

Bivalent messenger RNA (mRNA) vaccines, designed to combat emerging severe acute respiratory syndrome coronavirus 2 (SARS-CoV-2) variants, incorporate ancestral strains and a new variant. Our study assessed the immune response in previously vaccinated individuals of the Swiss HIV Cohort Study (SHCS) and the Swiss Transplant Cohort Study (STCS) following bivalent mRNA vaccination.

**Methods:**

Eligible SHCS and STCS participants received approved bivalent mRNA SARS-CoV-2 vaccines (mRNA-1273.214 or BA.1-adapted BNT162b2) within clinical routine. Blood samples were collected at baseline, 4 weeks, 8 weeks, and 6 months postvaccination. We analyzed the proportion of participants with anti-spike protein antibody response ≥1642 units/mL (indicating protection against SARS-CoV-2 infection), and in a subsample T-cell response (including mean concentrations), stratifying results by cohorts and population characteristics.

**Results:**

In SHCS participants, baseline anti-spike antibody concentrations ≥1642 units/mL were observed in 87% (96/112), reaching nearly 100% at follow-ups. Among STCS participants, 58% (35/60) had baseline antibodies ≥1642 units/mL, increasing to 80% at 6 months. Except for lung transplant recipients, all participants showed a 5-fold increase in geometric mean antibody concentrations at 4 weeks and a reduction by half at 6 months. At baseline, T-cell responses were positive in 96% (26/27) of SHCS participants and 36% (16/45) of STCS participants (moderate increase to 53% at 6 months). Few participants reported SARS-CoV-2 infections, side-effects, or serious adverse events.

**Conclusions:**

Bivalent mRNA vaccination elicited a robust humoral response in individuals with human immunodeficiency virus (HIV) or solid organ transplants, with delayed responses in lung transplant recipients. Despite a waning effect, antibody levels remained high at 6 months and adverse events were rare.

***Clinical Trials Registration***. NCT04805125.

## Teaser

Kidney transplant recipients and people with HIV had a robust cellular and humoral response after receiving bivalent SARS-CoV-2 vaccines, lasting for 6 months, and adverse events were rare. Lung transplant recipients mounted a delayed and insufficient immune response.

Coronavirus disease 2019 (COVID-19) vaccines have substantially altered the course of the severe acute respiratory syndrome coronavirus 2 (SARS-CoV-2) pandemic by preventing an estimated 20 million deaths within the first year of vaccination programs [1]. According to the World Health Organization (WHO), over 13 billion vaccine doses have been administered worldwide [2]. In the European Union and the United States of America the by far most commonly used COVID-19 vaccines are the messenger RNA (mRNA) vaccines BNT162b2 (Comirnaty) produced by Pfizer-BioNTech (approximately 1 billion doses) and mRNA-1273 (Spikevax) produced by Moderna (approximately 400 million doses) [3, 4]. Both vaccines were tested in large randomized clinical trials among the general population where they have proven to be safe and effective in terms of preventing COVID-19 infections [5, 6].

Evidence on the immune response to SARS-CoV-2 vaccines among people with compromised immune function is of crucial importance for these frail patient populations, which were underrepresented or excluded from the vaccines licensing trials [5, 6]. For these reasons, we have established the Corona Vaccine Trial Platform (COVERALL) [7, 8], nested into the Swiss HIV Cohort Study (SHCS) [9] and the Swiss Transplant Cohort Study (STCS) [10], to investigate immune response and vaccine safety in immunocompromised hosts. COVERALL-1, a randomized trial, found comparable antibody responses after basic immunization with mRNA-1273 by Moderna and BNT162b2 by Pfizer-BioNTech, with solid organ transplant (SOT) recipients exhibiting lower responses than people with HIV (PWH) [11]. COVERALL-2, an observational extension study, revealed similar outcomes for booster vaccines (mRNA-1273 vs BNT162b2), showing a substantial increase in antibody response among SOT recipients compared to basic immunization [12, 13]. Moreover, lower CD4 cell counts and ongoing HIV-1 replication in PWH were associated with diminished immune responses [14].

With the rapid decrease in COVID-19 vaccine effectiveness against emerging variants [15–21], bivalent vaccines incorporating Omicron BA.1 spike mRNA alongside the original wildtype mRNA (mRNA-1273.214 from Moderna and BA.1-adapted BNT162b2 from Pfizer-BioNTech) were approved by Swiss authorities in August and October 2022 [22, 23]. In response, the Swiss Federal Office of Public Health recommended these bivalent vaccines for the adult population, prioritizing high-risk individuals and health professionals [24]. Consequently, we conducted COVERALL-3 to evaluate the safety and immune response, including T-cell response, of these bivalent SARS-CoV-2 vaccines over 6 months in previously vaccinated individuals from the SHCS and the STCS, who exhibited varying levels of immunosuppression.

## METHODS

### Study Design

COVERALL-3 was a prospective, longitudinal, multicentric observational study. The study was approved by the ethics committee Nordwest- and Zentralschweiz, Switzerland (BASEC No. 2022-01760). The COVERALL platform was registered (https://clinicaltrials.gov/ct2/show/NCT04805125; registration 18 March 2021) and the full protocols for all COVERALL studies are available in the trial registry. Study data were collected using the REDCap electronic data capture database, which was set up for the COVERALL platform [8], including risk-based centralized data monitoring throughout the study.

### Participants

During routine cohort study visits, study participants were recruited by treating physicians at the University Hospitals Basel, Zurich, Bern, and Lausanne. Targeting individuals with varying levels of immunosuppression, we aimed to enroll participants of the following groups: (1) PWH with CD4 cell counts <350 cells/µL; (2) PWH with CD4 cell counts ≥350 cells/µL; (3) lung transplant recipients; and (4) kidney transplant recipients. SHCS and STCS participants were eligible for COVERALL-3 if they received the bivalent vaccines (mRNA-1273.214 or BA.1-adapted BNT162b2) according to local clinical guidelines as part of routine care, that is, with a minimum of 2 previous shots of either mRNA-1273 or BNT162b2. Of note, previous participation in COVERALL-1 and COVERALL-2 was not required (detailed eligibility criteria are in Supplementary Material 1).

### Data and Blood Sample Collection

Data collection commenced in October 2022 with the clinical routine rollout of both vaccines. Whole-blood samples (3 mL ethylenediamine tetraacetic acid [EDTA]) were obtained at baseline (up to 2 weeks before bivalent vaccination) and follow-up visits at 4 weeks (±1 week), 8 weeks (±2 weeks), and 6 months (±4 weeks). A subsample of participants provided an additional 8 mL heparinized blood at baseline, 4 weeks, and 6 months for anti-spike T-cell response measurement. Samples for the T-cell substudy were collected at the University Hospitals Basel and Zurich and were sent via courier within 6 hours at room temperature to the main laboratory in Zurich (Institute of Medical Virology). At baseline, study staff collected history of SARS-CoV-2 vaccination, seasonal flu vaccination, and treatment with SARS-CoV-2–specific monoclonal antibodies. Furthermore, anti-SARS-CoV-2 nucleocapsid (N) antibodies were assessed and all sociodemographic and clinical data (age, sex, history of cardiovascular or metabolic disease, CD4 T-cell counts, HIV viral load, and immunosuppressive therapy) were directly retrieved from the cohort databases. Clinical outcomes and serious adverse events were assessed at follow-up visits, with data on vaccine-specific side effects collected 4 weeks postvaccination.

### Laboratory Measurements

Pan-immunoglobulin (Ig) antibody response against the SARS-CoV-2 spike (S1) protein receptor binding domain was measured using the Elecsys Anti-SARS-CoV-2 S assay (Roche Diagnostics) at 2 different laboratories. The main laboratory in Zurich used 1:50 dilutions by default and further dilutions for samples with values above the measuring range until exact values were obtained. The second laboratory site at the University Hospital Basel conducted default repeat measurements of ≤1:10 diluted samples. Pan-Ig antibody response against the SARS-CoV-2 N antigen was assessed with the Elecsys Anti-SARS-CoV-2 assay. Spike protein-specific T-cell response was determined by the Quan-T-Cell SARS-CoV-2 interferon-γ (IFN-γ) release assay from EUROIMMUN (Medizinische Labordiagnostica) [25]. The kit included stimulation tubes for no T-cell stimulation (blank), specific T-cell stimulation (S1-based antigens; tube), and unspecific T-cell stimulation (stim). Data were analyzed based on manufacturer-defined criteria for negative (blank) and positive (stim) controls, with samples having nonvalid controls labelled as “nonevaluable”.

### Outcomes

We defined several immunological outcomes: the proportion of participants with an anti-spike protein (pan-Ig) antibody response of (1) ≥ 1642 units/mL, (2) ≥ 100 units/mL, and (3) ≥ 0.8 units/mL, at 4 weeks, 8 weeks, and 6 months. Additionally, we assessed geometric mean concentrations of the anti-spike protein (pan-Ig) antibody response at these time points. Geometric mean concentrations were calculated as the anti-logs of the means and address anticipated right-skewness of the data. A value of ≥0.8 units/mL was considered a positive response according to the manufacturer's instructions. The cutoff of 100 units/mL was chosen to allow a comparison with previous COVERALL studies [11, 12], and the cutoff of 1642 units/mL was used as a surrogate marker to predict protection against SARS-CoV-2 infection with Omicron strains based on Chen et al [26]. Of note, 1689 binding antibody units (BAU)/mL as described as a protective concentration by Chen et al are equal to 1642 units/mL using the WHO standardization formula (1 unit/mL = 0.972 × BAU/mL) based on the Elecsys Anti-SARS-CoV-2 S assay [26].

For the T-cell subsample, we determined spike protein-specific T-cell responses using IFN-γ concentrations according to the manufacturer’s instructions (positive, >200 mIU/mL; borderline, 100–200 mIU/mL; negative, <100 mIU/mL) [25]. Additionally, we quantitatively assessed the response on an IFN-γ concentration scale.

Clinical outcomes were the proportion of patients who reported a polymerase chain reaction (PCR) or antigen test-confirmed (1) asymptomatic and (2) symptomatic SARS-CoV-2 infection, as well as (3) severe COVID-19 requiring hospitalization or resulting in death.

Safety outcomes encompassed (1) any local symptom (such as redness or swelling, or prolonged pain at injection site) impeding normal daily activities; (2) any systemic symptom (eg, fever, generalized muscle or joint pain) impeding normal daily activities; and (3) any vaccine-related symptom prompting contact with a physician within the initial 7 days postvaccination.

### Data Analysis

All conducted analyses were of exploratory nature, without a priori power calculation. In the primary analysis set, all patients were included regardless of the time window. As a sensitivity analysis, we only included patients with results available within the prespecified time window (“strict time window”; i.e., baseline sample maximum up to 2 weeks before vaccination, 4 weeks after vaccination ±1 week, 8 weeks ±2 weeks, and 6 months ±4 weeks).

Descriptive statistics were used to present immunological and safety outcomes (ie, frequencies, percentages, and 95% confidence intervals [CI]).

For the geometric mean concentration of antibody response, we excluded samples analyzed at the University Hospital of Basel due to absence of reruns for antibody concentrations >2500 units/mL. Results are presented stratified by cohort study (SHCS vs STCS), population of interest (SHCS participants with CD4 below vs above 350 cells/µL; STCS participants receiving a kidney vs lung transplant), and prior immune response status (participants with any evidence of immunization [positive nucleocapsid antibodies or SARS-CoV-2 vaccination in the past 6 months] vs participants with no evidence of prior immunization) and not by vaccine type, given significant baseline characteristics differences. All data processing, graphing, and statistical analyses were performed using R Project for Statistical Computing (version 4.1.3) [27].

## RESULTS

### Baseline Characteristics of Participants

Between October 2022 and January 2023, 174 participants were enrolled (112 SHCS; 62 STCS) with 58% (101/174) receiving mRNA-1273.214 and 42% (73/174) receiving BA.1-adapted BNT162b2 vaccines (Table 1). The majority of participants were male (78.7% overall, 64.5% in STCS, and 86.6% in SHCS; Table 1). The overall median age was 56 years (interquartile range [IQR], 45–64 years), with 59 years (IQR, 47–65 years) in the STCS and 55 years (IQR, 44–63 years) in the SHCS (Table 1). Among SHCS participants, 82% (92/112) had CD4 cell counts of 350 cells/µL or above, with the majority (93.8%, 105/112) having a suppressed viral load (ie, <50 copies/mL). One-third of the STCS participants were kidney transplant recipients (35%, 22/62), while two-thirds received a lung transplant (65%, 40/62). Considering all participants, at baseline 59% (102/174) had a reactive antibody test to the nucleocapsid protein suggesting a previous SARS-CoV-2 infection (44% [27/62] among STCS and 67% [75/112] among SHCS participants). Most participants had received 3 (87%, 151/174) or 4 (10%, 17/174) doses of the respective monovalent vaccines before receiving the bivalent vaccine dose and only 4% (7/174) have received a SARS-CoV-2 vaccine in the 6 months prior receiving the bivalent vaccine. None of the SHCS participants had received SARS-CoV-2–specific monoclonal antibodies within the 6 months prior to vaccination. From the STCS, 3 kidney transplant (14%, 3/22) and 1 lung transplant recipients (3%, 1/40) had received SARS-CoV-2–specific monoclonal antibodies (all sotrovimab). Most STCS participants were receiving intensive immunosuppressive therapy, defined as more than 2 regimens (87%, 54/62), whereas only 13% (8/62) were taking a less-intense regimen (Table 1).

**Table 1.** Baseline Characteristics of Participants Before Receiving a Bivalent mRNA SARS-CoV-2 Vaccine

	People with HIV			Solid Organ Transplant Recipients			Total
Characteristic	CD4 < 350 Cells/µL (n = 20)	CD4 ≥ 350 Cells/µL (n = 92)	All (n = 112)	Kidney (n = 22)	Lung (n = 40)	All (n = 62)	(n = 174)
Bivalent mRNA SARS-CoV-2 vaccine type received							
mRNA-1273.214 vaccine, Moderna	6 (30.0)	55 (59.8)	61 (54.5)	11 (50.0)	29 (72.5)	40 (64.5)	101 (58.0)
BA.1-adapted BNT162b2 vaccine, Pfizer-BioNTech	14 (70.0)	37 (40.2)	51 (45.5)	11 (50.0)	11 (27.5)	22 (35.5)	73 (42.0)
Median age, y (IQR)	57 (44–62)	55 (44–63)	55 (44–63)	61 (48–65)	57 (48–64)	59 (47–65)	56 (45–64)
Sex							
Male	19 (95.0)	78 (84.8)	97 (86.6)	17 (77.3)	23 (57.5)	40 (64.5)	137/174 (78.7)
Female	1 (5.0)	14 (15.2)	15 (13.4)	5 (22.7)	17 (42.5)	22 (35.5)	37/174 (21.3)
Antibody test to the nucleocapsid protein							
Nonreactive	6 (30.0)	30 (32.6)	36 (32.1)	12 (54.5)	21 (52.5)	33 (53.2)	69/174 (39.7)
Reactive	14 (70.0)	61 (66.3)	75 (67.0)	10 (45.5)	17 (42.5)	27 (43.5)	102/174 (58.6)
Missing	0	1 (1.1)	1 (0.9)	0	2 (5.0)	2 (3.2)	3/174 (1.7)
History of cardiovascular disease or metabolic syndrome	8 (40.0)	31 (33.7)	39 (34.8)	22 (100.0)	34 (85.0)	56 (90.3)	95/174 (54.6)
Previous SARS-CoV-2 vaccine in the past 6 months	1 (5.0)	0 (0.0)	1 (0.9)	2 (9.1)	4 (10.0)	6 (9.7)	7/174 (4.0)
Number of previously received SARS-CoV-2 vaccines							
2	0 (0.0)	2 (2.2)	2 (1.8)	2 (9.1)	1 (2.5)	3 (4.8)	5/174 (2.9)
3	18 (90.0)	89 (96.7)	107 (95.5)	18 (81.8)	26 (65.0)	44 (71.0)	151/174 (86.8)
4	2 (10.0)	1 (1.1)	3 (2.7)	2 (9.1)	12 (30.0)	14 (22.6)	17/174 (9.8)
5	0 (0.0)	0 (0.0)	0 (0.0)	0 (0.0)	1 (2.5)	1 (1.6)	1/174 (0.6)
Seasonal flu vaccine 2022/2023 received	9 (45.0)	60 (65.2)	69 (61.6)	15 (68.2)	25 (62.5)	40 (64.5)	109/174 (62.6)
Suppressed HIV viral load^a,b^	18 (90.0)	87 (94.6)	105 (93.8)	…	…	…	105/112 (93.8)
Current immunosuppressive therapy^c^							
Less intense, ≤2-drug regimen^d^	…	…	…	6 (27.3)	2 (5.0)	8 (12.9)	8/62 (12.9)
Intense, 3- or 4-drug regimen^d^	…	…	…	16 (72.7)	38 (95.0)	54 (87.1)	54/62 (87.1)
Median days since transplant (IQR)^c^	…	…	…	671 (245–830)	1219 (591–2910)	859 (462–2881)	859 (462–2881)
SARS-CoV-2–specific monoclonal antibodies received within the last 6 months	0	0	0	3 (13.6)	1 (2.5)	4 (6.5)	4/174 (2.3)

Data are No. (%) except where indicated.

Abbreviations: HIV, human immunodeficiency virus; IQR, interquartile range; SARS-CoV-2, severe acute respiratory syndrome coronavirus 2.

^a^Only considering participants from the Swiss HIV Cohort Study.

^b^Suppressed HIV viral load defined as <50 copies/mL.

^c^Only considering participants from the Swiss Transplant Cohort Study.

^d^Intense treatment defined as triple or quadruple immunosuppressive regimen versus less-intense immunosuppressive therapy defined as dual immunosuppressive regimen.

Baseline stratification by vaccine type revealed notable differences (Supplementary Table 1). Specifically, 65% (40/62) of STCS participants received the mRNA-1273.214 vaccine, while only 54% (61/112) of SHCS participants did so. Of the 40 STCS participants receiving mRNA-1273.214 vaccine, 73% (29/40) were lung transplant recipients compared to 50% (11/22) in the BA.1-adapted BNT162b2 group. Further baseline characteristics of participants stratified by vaccine type and cohort study are presented in Supplementary Table 2.

### Antibody Status and Response

At baseline, 87% (96/112) of SHCS participants had anti-spike antibody concentrations ≥1642 units/mL, increasing to 97% (95% CI, 94%–100%; 103/106) at 4 weeks, 98% (95% CI, 95%–100%; 99/101) at 8 weeks, and 96% (95% CI, 92%–100%; 96/100) at 6 months (Table 2). Among STCS participants, 58% (35/60) had anti-spike antibody concentrations ≥1642 units/mL at baseline, reaching 75% (95% CI, 64%–87%; 43/57) at 4 weeks, 74% (95% CI, 63%–85%; 43/58) at 8 weeks, and 80% (95% CI, 70%–91%; 45/56) at 6 months (Table 2).

**Table 2.** Antibody Status Before and After Vaccination With Bivalent mRNA SARS-CoV-2 Vaccines in Participants With Different Levels of Immunosuppression, Measured With the Elecsys Anti-SARS-CoV-2 S Assay From Roche

	People with HIV			Solid Organ Transplant Recipients		
Antibody status	CD4 < 350 Cells/µL (n = 20)	CD4 ≥ 350 Cells/µL (n = 92)	All (n = 112)	Kidney (n = 22)	Lung (n = 40)	All (n = 62)
Baseline						
Antibody response, cutoff ≥1642 units/mL	75 (15/20)	89 (81/91)	87 (96/111)	77 (17/22)	47 (18/38)	58 (35/60)
Antibody response, cutoff ≥100 units/mL	90 (18/20)	100 (91/91)	98 (109/111)	100 (22/22)	84 (32/38)	90 (54/60)
Antibody response, cutoff ≥0.8 units/mL	100 (20/20)	100 (91/91)	100 (111/111)	100 (22/22)	92 (35/38)	95 (57/60)
Geometric mean concentration (IQR)^a^	4398 (1406–13 764)	8992 (6899–11 721)	7955 (5965–10 608)	5102 (1819–14 310)	1226 (491–3061)	1865 (907–3836)
4 weeks follow-up						
Antibody response, cutoff ≥1642 units/mL	85 (69–100) 17/20	100 (NA) 86/86	97 (94–100) 103/106	95 (86–100) 20/21	64 (49–80) 23/36	75 (64–87) 43/57
Antibody response, cutoff ≥100 units/mL	95 (85–100) 19/20	100 (NA) 86/86	99 (97–100) 105/106	100 (NA) 21/21	83 (71–96) 30/36	89 (82–97) 51/57
Antibody response, cutoff ≥0.8 units/mL	100 (NA) 20/20	100 (NA) 86/86	100 (NA) 106/106	100 (NA) 21/21	92 (83–100) 33/36	95 (89–100) 54/57
Geometric mean concentration (IQR)^a^	32 342 (12 638–82 766)	53 461 (44 474–62 264)	48 837 (39 209–60 830)	23 956 (8098–70 872)	3108 (1137–8499)	5772 (2556–13 030)
8 weeks follow-up						
Antibody response, cutoff ≥1642 units/mL	94 (84–100) 17/18	99 (96–100) 82/83	98 (95–100) 99/101	95 (87–100) 21/22	61 (45–77) 22/36	74 (63–85) 43/58
Antibody response, cutoff ≥100 units/mL	100 (NA) 18/18	100 (NA) 83/83	100 (NA) 101/101	100 (NA) 22/22	89 (79–99) 32/36	93 (87–100) 54/58
Antibody response, cutoff ≥0.8 units/mL	100 (NA) 18/18	100 (NA) 83/83	100 (NA) 101/101	100 (NA) 22/22	92 (83–100) 33/36	95 (89–100) 55/58
Geometric mean concentration (IQR)^a^	27 852 (15 583–49 781)	36 581 (29 366–45 568)	34 939 (28 505–42 826)	23 462 (7651–71 947)	4216 (1631–10 898)	7208 (3362–15 454)
6 months follow-up						
Antibody response, cutoff ≥1642 units/mL	82 (64–100) 14/17	99 (97–100) 82/83	96 (92–100) 96/100	95 (86–100) 20/21	71 (56–86) 25/35	80 (70–91) 45/56
Antibody response, cutoff ≥100 units/mL	100 (NA) 17/17	100 (NA) 83/83	100 (NA) 100/100	100 (NA) 21/21	91 (82–100) 32/35	95 (89–100) 53/56
Antibody response, cutoff ≥0.8 units/mL	100 (NA) 17/17	100 (NA) 83/83	100 (NA) 100/100	100 (NA) 21/21	94 (86–100) 33/35	96 (92–100) 54/56
Geometric mean concentration (IQR)^a^	12 426 (5846–26 412)	18 088 (14 577–22 446)	17 036 (13 774–21 071)	12 899 (4535–36 695)	5722 (3142–10 421)	7438 (4459–12 407)

Data are presented as “% (No./No)” at baseline and “% (95% confidence interval) No./No” at follow-up.

Abbreviations: IQR, interquartile range; NA, not applicable.

^a^Excluding study samples from the University Hospital Basel center as the measuring range was up to 2500 units/mL (ie, patients with HIV: n = 2 with CD4 < 350 cells/µL CD4; n = 4 with CD4 ≥ 350 cells/µL; n = 12 kidney transplant recipients; and n = 14 lung transplant recipients).

**Figure 1.**
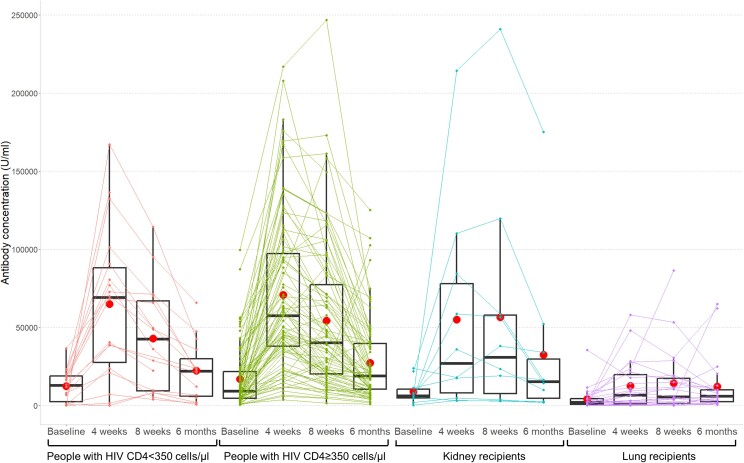
Antibody concentrations before and after vaccination with bivalent mRNA SARS-CoV-2 vaccines in PWH (stratified by CD4 cell counts below and above 350 cells/µL) and solid organ transplant recipient (stratified by kidney and lung recipients), measured with the Elecsys Anti-SARS-CoV-2 S assay. Red dots indicate mean values; boxplots indicate median and interquartile range, whiskers indicate minimum and maximum (excluding outliers). The University Hospital Basel center did not perform reruns with further dilutions if the antibody concentrations were >2500 units/mL. Hence, in order not to distort the results, all samples from the University Hospital of Basel were excluded. Abbreviations: mRNA, messenger RNA; PWH, people with human immunodeficiency virus; SARS-CoV-2, severe acute respiratory syndrome coronavirus 2.

All groups except for lung transplant recipients exhibited a 5-fold increase in geometric mean antibody concentrations at 4 weeks (ie, SHCS participants with CD4 < 350 cells/µL, baseline 4398 [IQR, 1406–13 764]; 4 weeks 32 342 [IQR, 12 638–82 766]; SHCS participants with CD4 ≥ 350 cells/µL, baseline 8992 [IQR, 6899–11 721]; 4 weeks 53 461 [IQR, 44 474–62 264]; kidney transplant recipients, baseline 5102 [IQR, 1819–14 310], 4 weeks 23 956 [IQR, 8098–70 872]; lung transplant recipients, baseline 1226 [95% CI, 491–3061], 4 weeks 3108 [95% CI, 1137–8499]; Table 2 and Figure 1). At 6 months, geometric mean antibody concentrations decreased to 12 426 (IQR, 5846–26 412) for SHCS participants with CD4 < 350 cells/µL, to 18 088 (IQR, 14 577–22 446) in SHCS participants with CD4 ≥ 350 cells/µL, to 12 899 (IQR, 4535–36 695) in kidney transplant recipients and increased to 5722 (IQR, 3142–10 421) for lung transplant recipients (Table 2 and Figure 1). For other cutoffs (≥100 units/mL and ≥0.8 units/mL), nearly all participants had reached these levels already at baseline (Table 2). The sensitivity analysis using the strict time window yielded similar findings (Supplementary Table 3).

The response dynamic in terms of anti-spike antibody concentration ≥1642 units/mL was similar among SHCS participants receiving the mRNA-1273.214 (87% at baseline; 96% at 4 weeks, 8 weeks, and 6 months) and those receiving the BA.1-adapted BNT162b2 (86% at baseline; 98% [95% CI, 94%–100%] at 4 weeks; 100% at 8 weeks; and 96% [95% CI, 90–100] at 6 months; Supplementary Table 4). A higher proportion of STCS participants vaccinated with BA.1-adapted BNT162b2 had antibody concentrations of ≥1642 units/mL already at baseline (75%) compared to those vaccinated with mRNA-1273.214 (53%) (Supplementary Table 5). Subsequently, across both vaccine groups, 15% more participants had an anti-spike antibody response of ≥1642 units/mL at 4 weeks (BA.1-adapted BNT162b2, 89% [95% CI, 76%–100%]; mRNA-1273.214, 68% [95% CI, 54%–83%]) and remained similarly high at 6 months (BA.1-adapted BNT162b2, 95% [95% CI, 85%–100%]; mRNA-1273.214, 72% [95% CI, 58%–87%]; Supplementary Table 5). Further stratification showed that baseline antibodies were higher amongst individuals with evidence of prior immunization, but subsequent response dynamic was similar across both groups (Supplementary Table 6). Details about the 17 cases who did not mount a sufficient humoral immune response at 8 weeks are provided in Supplementary Table 7.

### T-Cell Status and Response

**Figure 2.**
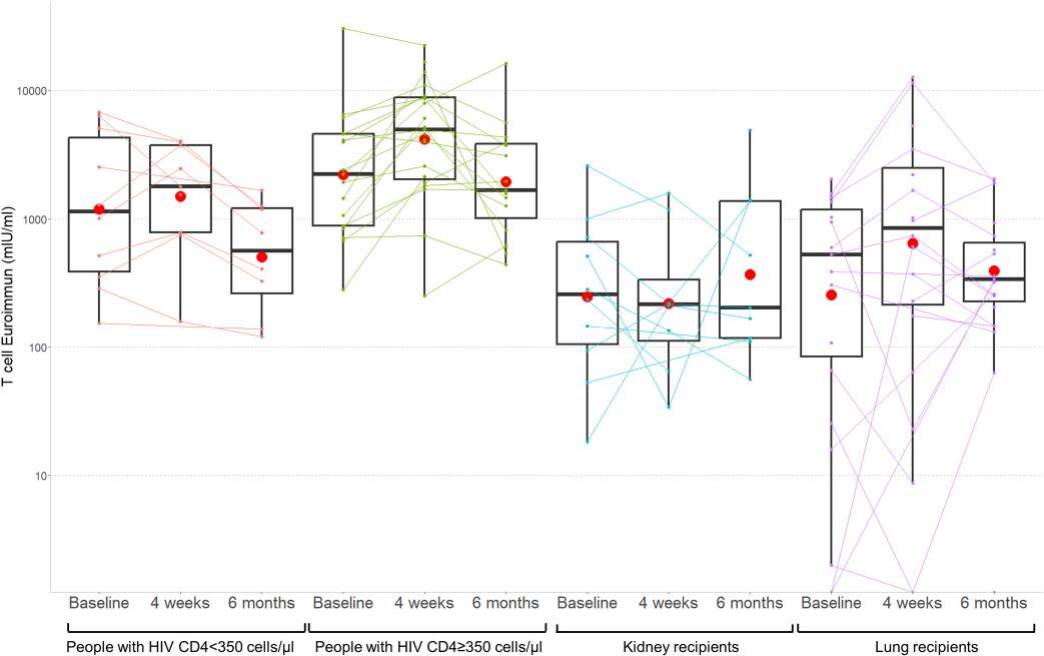
T-cell geometric mean concentration before and after vaccination with bivalent mRNA SARS-CoV-2 vaccines in PWH (stratified by CD4 cell counts below and above 350 cells/µL) and solid organ transplant recipients (stratified by kidney and lung recipients), measured with the interferon-γ release assay. Red dots indicate mean values, boxplots indicate median and interquartile range; whiskers indicate minimum and maximum (excluding outliers). Participants with a nonevaluable T-cell response were excluded. Abbreviations: mRNA, messenger RNA; PWH, people with human immunodeficiency virus; SARS-CoV-2, severe acute respiratory syndrome coronavirus 2.

From 81 of the 174 participants, T-cell response was assessed after vaccination with mRNA-1273.214 (n = 54) or BA.1-adapted BNT162b2 (n = 27; see baseline characteristics in Supplementary Table 8). Among SHCS participants, all had evaluable results, with 96% (26/27) having a positive status and 1 (4%, 1/27) having a borderline status at baseline. The geometric mean concentration increased for both SHCS groups at 4 weeks (ie, CD4 < 350, baseline 1193 [IQR, 448–3179]; 4 weeks 1500 [IQR, 664–3386]; CD4 ≥ 350, baseline 2202 [IQR, 1234–3931]; 4 weeks 4189 [IQR, 2671–6571]) and decreased at 6 months (CD4 < 350, 507 [IQR, 218–1180]; CD4 ≥ 350, 1939 [IQR, 1123–3347]; Table 3 and Figure 2).

**Table 3.** T-Cell Status Before and After Vaccination With Bivalent mRNA SARS-CoV-2 Vaccines in a Subsample of Participants With Different Levels of Immunosuppression, Measured With the Interferon-γ Release Assay From Euroimmun

	People with HIV			Solid Organ Transplant Recipients		
T-cell status	CD4 < 350 Cells/µL (n = 11)	CD4 ≥ 350 Cells/µL (n = 24)	All (n = 35)	Kidney (n = 11)	Lung (n = 35)	All (n = 46)
Baseline						
Positive	90; 9/10	100; 17/17	96; 26/27	55; 6/11	29; 10/34	36; 16/45
Borderline	10; 1/10	0; 0/17	4; 1/27	9; 1/11	3; 1/34	4; 2/45
Negative	0; 0/10	0; 0/17	0; 0/27	27; 3/11	18; 6/34	20; 9/45
Not evaluable	0; 0/10	0; 0/17	0; 0/27	9; 1/11	50; 17/34	40; 18/45
Geometric mean concentration (IQR)^a^	1193 (448–3179) n = 10	2202 (1234–3931) n = 17	1755 (1077–2859) n = 27	247 (86–710) n = 10	45 (3–674) n = 17	85 (16–464) n = 27
4 weeks follow-up						
Positive	73 (46–99) 8/11	96 (88–100) 24/24	89 (78–99) 31/34	50 (19–81) 5/10	36 (20–53) 12/33	40 (25–54) 17/43
Borderline	9 (0–26) 1/11	0 (NA) 0/24	3 (0–8) 1/35	10 (0–29) 1/10	3 (0–9) 1/33	5 (0–11) 2/43
Negative	0 (NA) 0/11	0 (NA) 0/24	0 (NA) 0/35	20 (0–45) 2/10	21 (7–35) 7/33	21 (9–33) 9/43
Not evaluable	18 (0–41) 2/11	4 (0–12) 1/24	9 (0–18) 3/35	20 (0–45) 2/10	39 (23–56) 13/33	35 (21–49) 15/43
Geometric mean concentration (IQR)^a^	1500 (664–3386) n = 9	4189 (2671–6571) n = 23	3138 (2088–4718) n = 32	221 (74–659) n = 8	28 (1–642) n = 20	51 (5–467) n = 28
6 months follow-up						
Positive	75 (45–100) 6/8	100 (NA) 15/15	91 (80–100) 21/23	50 (19–81) 5/10	54 (35–72) 15/28	53 (37–69) 20/38
Borderline	25 (0–55) 2/8	0 (NA) 0/15	9 (0–20) 2/23	30 (2–58) 3/10	10 (0–22) 3/28	16 (4–27) 6/38
Negative	0 (NA) 0/8	0 (NA) 0/15	0 (NA) 0/23	10 (0–29) 1/10	7 (0–17) 2/28	8 (0–16) 3/38
Not evaluable	0 (NA) 0/8	0 (NA) 0/15	0 (NA) 0/23	10 (0–29) 1/10	29 (12–45) 8/28	24 (10–37) 9/38
Geometric mean concentration (IQR)^a^	507 (208–1180) n = 8	1939 (1123–3347) n = 15	1216 (733–2017) n = 23	368 (118–1152) n = 9	185 (36–962) n = 20	229 (72–724) n = 29

Data are presented as “% (No./No)” at baseline and “% (95% confidence interval) No./No” at follow-up, except where indicated.

Abbreviations: IQR, interquartile range; NA, not applicable.

^a^Excluding not evaluable patients.

Among STCS participants, 36% (16/45) had a positive status at baseline and 40% (18/45) were not evaluable due to a nonvalid control (primarily in lung transplant recipients; 50% [17/34] not evaluable; Table 3). The proportion with a positive reaction remained relatively low at 4 weeks (40%, 17/43) and at 6 months (53%, 20/38) after vaccination. On a quantitative scale, geometric mean concentrations were low at baseline (kidney recipients, 247 [IQR, 86–710]; lung recipients, 45 [IQR, 3–674]). After vaccination, they remained low at 4 weeks (kidney recipients, 221 [IQR, 74–659]; lung recipients, 28 [IQR, 1–642]) and 6 months (kidney recipients, 368 [IQR, 118–1152]; lung recipients, 185 [IQR, 36–962]; Table 3 and Figure 2). The proportion of not evaluable samples among SOT recipients was 35% at 4 weeks and 24% at 6 months. The analyses using the strict time window and stratified by evidence of previous immunization are presented in Supplementary Tables 9 and 10, showing similar response results.

### Clinical Outcomes

Until 6 months of follow-up, 8 symptomatic antigen- or PCR-confirmed SARS-CoV-2 infections occurred (Table 4). Among the 8 infected participants, 1 was from the SHCS and 7 from the STCS (1 kidney transplant recipient, 6 lung transplant recipients), 7 had anti-spike antibody concentrations ≥1642 units/mL, and 3 a reactive T-cell test at the study visit before acquiring a SARS-CoV-2 infection. Details about the 8 clinical cases are provided in Supplementary Table 11. No severe COVID-19 cases (requiring hospitalization or leading to death) occurred. Four serious adverse events were reported, including 1 death in an SHCS participants due to cancer, and 3 STCS participants hospitalized for infectious diseases, all not related to COVID-19. The local investigator judged that all 4 serious adverse events were not related to the vaccination. Systemic and local symptoms limiting daily activities occurred infrequently (7%–8% with mRNA-1273.214 and 4%–8% with BA.1-adapted BNT162b2; Table 4).

**Table 4.** Clinical Outcomes After Vaccination With Bivalent mRNA SARS-CoV-2 Vaccines in Participants With Different Levels of Immunosuppression

	mRNA-1273.214, Moderna			BA.1-Adapted BNT162b2, Pfizer-BioNTech			Total
Clinical outcomes and adverse events	SHCS (n = 61)	STCS (n = 40)	All (n = 101)	SHCS (n = 51)	STCS (n = 22)	All (n = 73)	(n = 174)
SARS-CoV-2 infection 4 weeks^a^	0 (0/55)	3 (1/40)	1 (1/91)	0 (0/41)	5 (1/21)	2 (1/60)	1 (2/151)
SARS-CoV-2 infection 4–8 weeks	0 (0/54)	0 (0/40)	0 (0/95)	0 (0/47)	0 (0/19)	0 (0/66)	0 (0/161)
SARS-CoV-2 infection 8 week–6 monhts^b^	0 (0/54)	13 (5/40)	5 (5/95)	2 (1/47)	0 (0/19)	1 (1/66)	4 (6/161)
Severe COVID-19 disease, requiring hospitalization or leading to death	0 (0/61)	0 (0/40)	0 (0/101)	0 (0/51)	0 (0/22)	0 (0/73)	0 (0/174)
Serious adverse events	0 (0/61)	8 (3/40)	3 (3/101)	0 (0/51)	5 (1/22)	1 (1/73)	2 (4/174)
Any local symptoms limiting continuation of normal daily activities during the first 7 days	12 (7/60)	0 (0/40)	7 (7/100)	5 (5/50)	5 (1/22)	8 (6/72)	8 (13/172)
Any systemic symptoms limiting continuation of normal daily activities during the first 7 days	12 (7/60)	3 (1/40)	8 (8/100)	6 (3/50)	0 (0/22)	4 (3/72)	6 (11/172)
Any vaccine related symptom leading to contacting a physician during the first 7 days	0 (0/60)	0 (0/40)	0 (0/100)	0 (0/50)	0 (0/22)	0 (0/72)	0 (0/172)

Data are % (No. with outcome/No. with assessed outcome).

Abbreviations: COVID-19, coronavirus disease 2019; SARS-CoV-2, severe acute respiratory syndrome coronavirus 2; SHCS, Swiss HIV Cohort Study; STCS, Swiss Transplant Cohort Study.

^a^Both symptomatic with the following symptoms: cough, shortness of breath, muscle aches, sore throat.

^b^All symptomatic with the following symptoms: fever or chills, cough, shortness of breath, fatigue, muscle or body aches, headache, sore throat, congestion or runny nose, nausea, or vomiting.

## DISCUSSION

This study examined the antibody and T-cell response to bivalent SARS-CoV-2 vaccines (Moderna’s mRNA-1273.214 or Pfizer-BioNTech’s BA.1-adapted BNT162b2) in PWH and SOT recipients who had received at least 2 prior SARS-CoV-2 vaccine doses. Both bivalent vaccines increased the proportion of individuals achieving a humoral immune response of ≥1642 units/mL, suggesting protection from COVID-19, and at 6 months a waning effect was observed. However, lung transplant recipients showed a delayed and lower humoral response throughout. T-cell–mediated immune response among SHCS participants remained consistently high, while STCS participants showed low baseline activity, and only a minor increase at 6 months. Seven out of 8 breakthrough infections were observed among SOT recipients (5 lung and 2 kidney recipients); however, not a single case of severe COVID-19 disease, requiring hospitalization or leading to death, was observed. During the study period SARS-CoV-2 infections, side-effects, and serious events were rare. In immunocompetent individuals, the bivalent mRNA vaccines have demonstrated high immunogenicity against Omicron and Omicron sublineages [28], including effectiveness on clinical outcomes [20, 29]. However, limited data exist among immunocompromised individuals receiving these bivalent vaccines.

Similar to our findings, an observational cohort study on 48 PWH revealed a significant humoral response increase after bivalent mRNA vaccination, irrespective of CD4 cell count, while T-cell–mediated response remained unchanged [30]. This aligns with previous research indicating a robust T-cell response in PWH after 2 doses of SARS-CoV-2 vaccines, which remained stable after the third dose [31]. Earlier studies in the same setting (ie, COVERALL 1 and 2), also demonstrated solid immune response in PWH after basic immunization [11] and after a third vaccine dose [13].

SOT recipients are a critical population in terms of vaccine-induced immune response [32]. Unlike in PWH, 9% of kidney transplant recipients and 50% of lung transplant recipients had a nonevaluable T-cell response and among the evaluable ones, only a minor increase in T-cell response was observed, most likely due to the concurrent intense immunosuppressive therapy as shown in previous mRNA vaccine studies [33–35]. A study among kidney transplant recipients concluded that a better antibody response after receiving the bivalent vaccine was associated with a lower drug-related immunosuppression [36].

A recent study explored cell-mediated responses against the BA.4/BA.5 spike receptor-binding domain at baseline and 2 weeks after the mRNA-based bivalent vaccination among 30 kidney or liver transplant recipients and compared the immune responses against a healthy control group [37]. In contrast to our results, kidney transplant recipients had a significant increase in T-cell activity already at 2 weeks. A recent meta-analysis summarized the humoral immune response rates among SOT recipients, showing increased immunogenicity with booster vaccinations and weakest response among lung transplant recipients, in line with our findings [37]. Ninety-five percent of the lung transplant recipients in our study were taking a 3- or 4-drug intense immunosuppressive therapy versus 73% of the kidney transplant recipient participants. None of the included studies in the meta-analysis evaluated any bivalent vaccine. We are not aware of further evidence about immune response elicited by the mRNA-based bivalent vaccines in SOT or PWH. However, several studies have assessed the immunogenicity of the bivalent mRNA vaccines among immunocompetent but frail populations, such as nursing home residents, all showing a markedly increased humoral and cellular response postvaccination [38–40].

Previous studies observed significant differences in vaccine-induced immune response between different nonbivalent mRNA SARS-CoV-2 vaccines [32, 41]. Our study, while not designed for statistical power in comparing immune responses between the 2 bivalent vaccines, did not indicate any clear differences from descriptive analyses. Of note, such differences were often attributed to the varying concentration of mRNA in previous vaccines (e.g., the mRNA-1273 vaccine had 3-fold greater concentration than the BNT162b2 vaccine [32]).

Prior immunization has been shown to elicit an even stronger immune response across SARS-CoV-2 vaccine studies [30, 42, 43]. Nearly 60% of our participants had a positive nucleocapsid antibody test at baseline, suggestive of previous infection and 4% received a recent vaccination (within 6 months prior to baseline). Participants with evidence of prior immunization had higher baseline immune response, without marked differences in the immune response dynamic in the follow-up (Supplementary Tables 6 and 10). Our study has several limitations. First, we acknowledge that interpreting the anti-spike antibody level cutoff of 1642 units/mL requires caution. While at the beginning of the COVID-19 pandemic antibody responses were more straightforward to interpret, the emergence of several variants as well as repeated exposure to the virus have made the interpretation more difficult [44, 45]. Second, we do not have information on neutralizing antibodies for the new variants or data systematically collected on prior COVID-19 episodes except nucleocapsid antibodies as a proxy. Last, for the geometric mean concentration of the humoral response, we excluded all patients from the University Hospital Basel site (n = 32) due to variations in the local laboratory protocol for diluting high values compared to other laboratories.

In conclusion, this is the largest study on immune response in PWH and SOT recipients after receiving an mRNA-based bivalent COVID-19 vaccine. Despite a waning effect, antibody levels remained high at 6 months. SOT recipients, particularly lung transplant recipients, showed lower and delayed immune responses. Further research is required to understand the clinical implications of these immune response patterns.

## Supplementary Data

Supplementary materials are available at *The Journal of Infectious Diseases* online (http://jid.oxfordjournals.org/). Supplementary materials consist of data provided by the author that are published to benefit the reader. The posted materials are not copyedited. The contents of all supplementary data are the sole responsibility of the authors. Questions or messages regarding errors should be addressed to the author.

## Supplementary Material

jiae291_Supplementary_Data

## Notes

***Author contributions.*** A. Am., F. C., A. Au., A. G., N. D. L., H. C. B., H. F. G., I. A. A., M. B., and B. S. designed the study. K. K. developed the database structure and link between the SHCS and COVERALL study. F. C. conducted the statistical analyses. A. Am., F. C., C. M. S., and B. S. were responsible for monitoring. A. Am. and B. S. coordinated the study. A. L. E., D. L. B., P. A., M. P. S., B. H., M. P. O., O. M., C. B., M. M. S., R. H., D. D., M. T., N. J. M., A. R., H. F. G. were responsible for patient recruitment and follow-up at local centers. K. K., M. T. K., R. K., and I. A. A. managed the data matching with the cohort studies. A. Au. conducted all laboratory analyses. A. Am., F. C., A. Au., A. G., I. A. A., M. B., and B. S. interpreted the data. A. Am. and B. S. wrote the first draft of the manuscript. All authors read and approved the final version of the manuscript.

***Acknowledgments.*** We are grateful to all involved study staff at the local centers in Basel (Maria Pascarella, Louise Seiler), Zürich (Daniela Gsell, Katia Dettling, Laura Tschuor, Christina Grube, Flurina Brunschweiler, Christine Schneider, Andrea Wallensteiner, Daniel Götsch, Andrea Macedo, Esther Göldi, Alina Imoli), Bern (Manuela Correia da Silva Saúde, Daniela Hirter, Pia Scherler, Anna Rehbock), Lausanne (Angela Koutsokera), and the Institute of Medical Virology, University of Zürich. Most importantly, we thank all study participants.

***Members of the Swiss HIV Cohort Study.*** I. Abela, K. Aebi-Popp, A. Anagnostopoulos, M. Battegay, E. Bernasconi, D. L. Braun, H. C. Bucher, A. Calmy, M. Cavassini, A. Ciuffi, G. Dollenmaier, M. Egger, L. Elzi, J. Fehr, J. Fellay, H. Furrer, C. A. Fux, H. F. Günthard (President of the SHCS), A. Hachfeld, D. Haerry (Deputy of Positive Council), B. Hasse, H. H. Hirsch, M. Hoffmann, I. Hösli, M. Huber, D. Jackson-Perry (patient representatives), C. R. Kahlert (Chairman of the Mother and Child Substudy), L. Kaiser, O. Keiser, T. Klimkait, R. D. Kouyos, H. Kovari, K. Kusejko (Head of Data Centre), N. Labhardt, K. Leuzinger, B. Martinez de Tejada, C. Marzolini, K. J. Metzner, N. Müller, J. Nemeth, D. Nicca, J. Notter, P. Paioni, G. Pantaleo, M. Perreau, A. Rauch (Chairman of the Scientific Board), L. Salazar-Vizcaya, P. Schmid, R. Speck, M. Stöckle M (Chairman of the Clinical and Laboratory Committee), P. Tarr, A. Trkola, G. Wandeler, M. Weisser, S. Yerly.

***Members of the Swiss Transplant Cohort Study.*** Patrizia Amico, John-David Aubert, Vanessa Banz, Sonja Beckmann, Guido Beldi, Christoph Berger, Ekaterine Berishvili, Annalisa Berzigotti, Isabelle Binet, Pierre-Yves Bochud, Sanda Branca, Heiner C. Bucher, Emmanuelle Catana, Anne Cairoli, Yves Chalandon, Sabina De Geest, Olivier De Rougemont, Sophie De Seigneux, Michael Dickenmann, Joëlle Lynn Dreifuss, Michel Duchosal, Thomas Fehr, Sylvie Ferrari-Lacraz, Christian Garzoni, Déla Golshayan, Nicolas Goossens, Fadi Haidar, Jörg Halter, Dominik Heim, Christoph Hess, Sven Hillinger, Hans H Hirsch, Patricia Hirt, Linard Hoessly, Günther Hofbauer, Uyen Huynh-Do, Franz Immer, Michael Koller, Bettina Laesser, Frédéric Lamoth, Roger Lehmann, Alexander Leichtle, Oriol Manuel, Hans-Peter Marti, Michele Martinelli, Valérie McLin, Katell Mellac, Aurélia Merçay, Karin Mettler, Nicolas J Mueller, Ulrike Müller-Arndt, Beat Müllhaupt, Mirjam Nägeli, Graziano Oldani, Manuel Pascual, Jakob Passweg, Rosemarie Pazeller, Klara Posfay-Barbe, Juliane Rick, Anne Rosselet, Simona Rossi, Silvia Rothlin, Frank Ruschitzka, Thomas Schachtner, Stefan Schaub, Alexandra Scherrer, Aurelia Schnyder, Macé Schuurmans, Simon Schwab, Thierry Sengstag, Federico Simonetta, Susanne Stampf, Jürg Steiger, Guido Stirnimann, Ueli Stürzinger, Christian Van Delden, Jean-Pierre Venetz, Jean Villard, Julien Vionnet, Madeleine Wick, Markus Wilhelm, Patrick Yerly.

***Disclaimer***. The study protocol was reviewed by Moderna before conduct. The funders had no role in data collection and analysis, and preparation of the manuscript. Before submission, Moderna had the right to read the manuscript and make suggestions, but the study team was not obliged to accept suggestions and Moderna was not involved in the final decision to submit to the journal.

***Financial support*.** This work (COVERALL-3) was supported by Moderna. The setup of the study platform (ie, COVERALL) was funded by the Swiss National Science Foundation (grant number 31CA30_196245). The Swiss HIV Cohort Study (SHCS) and the Swiss Transplant Cohort Study (STCS) are funded by the Swiss National Science Foundation (grant numbers 177499 and 201369 to SHCS and 33CS30_177522 to STCS). The reagents required for the Elecsys Anti-SARS-CoV-2 S and Elecsys Anti-SARS-CoV-2 assays used at the Institute of Medical Virology in Zürich were provided by Roche. A. A. received his salary for the duration of this project from the Junior Research Fund of the University of Basel. C. M. S. received his salary for the duration of this project from the Swiss National Science Foundation (grant number 323530_221860) and the Janggen Pöhn Foundation.
