# Supplementary material for: Antibody and T-Cell Response to Bivalent Booster SARS-CoV-2 Vaccines in People With Compromised Immune Function: COVERALL-3 Study
Source: J Infect Dis. 2024 Jun 7;230(4):e847–59. doi: 10.1093/infdis/jiae291 (PMC11481330; doi:10.1093/infdis/jiae291)
Supplement: jiae291_Supplementary_Data [file jiae291_supplementary_data.docx]

**Appendix**

**Antibody and T-cell response to bivalent booster SARS-CoV-2 vaccines in people with compromised immune function (COVERALL-3)**

Alain Amstutz, Frédérique Chammartin, Annette Audigé, Anna L. Eichenberger, Dominique L. Braun, Patrizia Amico, Marcel P. Stoeckle, Barbara Hasse, Matthaios Papadimitriou-Olivgeris, Oriol Manuel, Cédric Bongard, Macé M. Schuurmans, René Hage, Dominik Damm, Michael Tamm, Nicolas J. Mueller, Andri Rauch, Huldrych F. Günthard, Michael T. Koller, Christof M. Schönenberger, Alexandra Griessbach, Niklaus D. Labhardt, Roger D. Kouyos, Alexandra Trkola, Katharina Kusejko, Heiner C. Bucher, Irene A. Abela, Matthias Briel, Benjamin Speich, and the Swiss HIV Cohort Study, and the Swiss Transplant Cohort Study

**Appendix 1:**

**Eligibility Criteria**

Inclusion criteria:

- - Patients with either a HIV infection or recipients of solid organs registered in the SHCS and STCS cohorts and signed informed consent form
  - Patients aged ≥18 years
  - Patients receiving a new bivalent (Wuhan/Omicron BA.1) mRNA SARS-CoV-2 vaccine in the frame of clinical routine, according to the treating physician
  - Provide consent for the COVERALL observational bivalent booster extension study (third sub-protocol)

Exclusion criteria:

- Acute symptomatic SARS-CoV-2 infection, influenza or other acute respiratory tract infection
- Any emergency condition requiring immediate hospitalization for any condition
- Known allergy or contra-indications for vaccines or any vaccine components or any other contraindication to receive the vaccine according to the treating physician
- Patients who did not receive the “basic immunization” SARS-CoV-2 vaccination (e.g. two doses of Spikevax from Moderna, two doses of Comirnaty from Pfizer-BioNtech).

**Table S1: Baseline characteristics stratified by bivalent SARS-CoV-2 vaccines received**

| **Characteristics** | **mRNA-1273.214 by Moderna** (n=101) | **BA.1–adapted BNT162b2 by Pfizer-BioNTech** (n=73) | **Total**  (n=174) |
| --- | --- | --- | --- |
| **Median age (IQR)** | 55 (44-63) | 59 (45-65) | 56 (45-64) |
| **Sex** |  |  |  |
| Male | 77/101 (76.2%) | 60/73 (82.2%) | 137/174 (78.7%) |
| Female | 24/101 (23.8%) | 13/73 (17.8%) | 37/174 (21.3%) |
| **Cohort** |  |  |  |
| SHCS | 61/101 (60.4%) | 51/73 (69.9%) | 112/174 (64.4%) |
| STCS | 40/101 (39.6%) | 22/73 (30.1%) | 62/174 (35.6%) |
| **Antibody test to the nucleocapsid protein** |  |  |  |
| Non-reactive | 42/101 (51.6%) | 27/73 (30.1%) | 69/174 (39.7%) |
| Reactive | 58/101 (57.4%) | 44/73 (60.3%) | 102/174 (58.6%) |
| Missing | 1/101 (1.0%) | 2/73 (2.7%) | 3/174 (1.7%) |
| **Previous SARS-CoV-2 vaccine in the past 6 months** | 2/101 (2.0%) | 5/73 (6.8%) | 7/174 (4.0%) |
| **History of cardiovascular disease or metabolic syndrome^a^** | 57/101 (56.4%) | 38/73 (52.1%) | 95/174 (54.6%) |
| **Number of previously received SARS-CoV-2 vaccines** |  |  |  |
| 2 | 3/101 (3.0%) | 2/73 (2.7%) | 5/174 (2.9%) |
| 3 | 89/101 (88.1%) | 62/73 (84.9%) | 151/174 (86.8%) |
| 4 | 8/101 (7.9%) | 9/73 (12.3%) | 17/174 (9.8%) |
| 5 | 1/101 (1.0%) | 0/73 (0%) | 1/174 (0.6%) |
| **Seasonal flu vaccine (2022/2023) received** | 62/101 (61.4%) | 47/73 (64.4%) | 109/174 (62.6%) |
| **CD4 cell count (cells/µL)^a^** |  |  |  |
| <350 | 6/61 (9.8%) | 14/51 (27.5%) | 20/112 (17.9%) |
| ≥350 | 55/61 (90.2%) | 37/51 (72.5%) | 92/112 (82.1%) |
| **Suppressed HIV viral load^ab^** | 57/61 (93.4%) | 48/51 (94.1%) | 105/112 (93.8%) |
| **Transplanted organ^c^** |  |  |  |
| Kidney transplant | 11/40 (27.5%) | 11/22 (50.0%) | 22/62 (35.5%) |
| Lung transplant | 29/40 (72.5%) | 11/22 (50.0%) | 40/62 (64.5%) |
| **Current Immunosuppressive therapy^c^** |  |  |  |
| Less intense (≤2 regimen)^d^ | 6/40 (15.0%) | 2/22 (9.1%) | 8/62 (12.9%) |
| Intense (3 or 4 regimen)^d^ | 34/40 (85.0%) | 20/22 (90.9%) | 54/62 (87.1%) |
| **Median days since transplant (IQR)^c^** | 1067 (665-2999) | 643 (287-2808) | 859 (462-2881) |
| **SARS-CoV-2 specific monoclonal antibodies received within the last 6 months** | 3/101 (3.0%) | 1/73 (1.4%) | 4/174 (2.3%) |

^a^Only considering participants from the Swiss HIV Cohort Study

^b^Suppressed HIV viral load defined as <50 copies/ml

^c^Only considering participants from the Swiss Transplant Cohort Study

^d^Intense treatment defined as triple or quadruple immunosuppressive regimen vs. less intense immunosuppressive therapy defined as dual immunosuppressive regimen

Abbreviations: IQR=Interquartile range; SHCS=Swiss HIV Cohort Study; STCT= Swiss Transplant Cohort Study

**Table S2: Baseline characteristics stratified by participating cohort study**

|  | **People living with HIV** | | | **Solid organ transplant recipients** | | |
| --- | --- | --- | --- | --- | --- | --- |
| **Characteristics** | **mRNA-1273.214 by Moderna** (n=61) | **BA.1–adapted BNT162b2 by Pfizer-BioNTech** (n=51) | **Total**  (n=112) | **mRNA-1273.214 by Moderna** (n=40) | **BA.1–adapted BNT162b2 by Pfizer-BioNTech** (n=22) | **Total**  (n=62) |
| **Median age (IQR)** | 54 (44-62) | 57 (45-64) | 55 (44-63) | 57 (48-64) | 61 (48-66) | 59 (47-65) |
| **Sex** |  |  |  |  |  |  |
| Male | 53 (86.9%) | 44 (86.3%) | 97 (86.6%) | 24 (60.0%) | 16 (72.7%) | 40 (64.5%) |
| Female | 8 (13.1%) | 7 (13.7%) | 15 (13.4%) | 16 (40.0%) | 6 (27.3%) | 22 (35.5%) |
| **Antibody test to the nucleocapsid protein** |  |  |  |  |  |  |
| Non-reactive | 18 (29.5%) | 18 (35.3%) | 36 (32.1%) | 24 (60.0%) | 9 (40.9%) | 33 (53.2%) |
| Reactive | 42 (68.9%) | 33 (64.7%) | 75 (67.0%) | 16 (40.0%) | 11 (50.0%) | 27 (43.5%) |
| Missing | 1 (1.6%) | 0 | 1 (0.9%) | 0 | 2 (9.1%) | 2 (3.2%) |
| **Previous SARS-CoV-2 vaccine in the past 6 months** | 0 (0.0%) | 1 (2.0%) | 1 (0.9%) | 2 (5.0%) | 4 (18.2%) | 6 (9.7%) |
| **History of cardiovascular disease or metabolic syndrome^a^** | 22 (36.1%) | 17 (33.3%) | 39 (34.8%) | 35 (87.5%) | 21 (95.5%) | 56 (90.3%) |
| **Number of previously received SARS-CoV-2 vaccines** |  |  |  |  |  |  |
| 2 | 1 (1.6%) | 1 (2.0%) | 2 (1.8%) | 2 (5.0%) | 1 (4.5%) | 3 (4.8%) |
| 3 | 60 (98.4%) | 47 (92.2%) | 107 (95.5%) | 29 (72.5%) | 15 (68.2%) | 44 (71.0%) |
| 4 | 0 (0.0%) | 3 (5.9%) | 3 (2.7%) | 8 (20.0%) | 6 (27.3%) | 14 (22.6%) |
| 5 | 0 (0.0%) | 0 (0.0%) | 0 (0.0%) | 1 (2.5%) | 0 (0.0%) | 1 (1.6%) |
| **Seasonal flu vaccine (2022/2023) received** | 39 (63.9%) | 30 (58.8%) | 69 (61.6%) | 23 (57.5%) | 17 (77.3%) | 40 (64.5%) |
| **CD4 cell count (cells/µL)^a^** |  |  |  |  |  |  |
| <350 | 6 (9.8%) | 14 (27.5%) | 20 (17.9%) | - | - | - |
| ≥350 | 55 (90.2%) | 37 (72.5%) | 92 (82.1%) | - | - | - |
| **Suppressed HIV viral load^ab^** | 57 (93.4%) | 48 (94.1%) | 105 (93.8%) | - | - | - |
| **Transplanted organ^c^** |  |  |  |  |  |  |
| Kidney transplant | - | - | - | 11 (27.5%) | 11 (50.0%) | 22 (35.5%) |
| Lung transplant | - | - | - | 29 (72.5%) | 11 (50.0%) | 40 (64.5%) |
| **Current Immunosuppressive therapy^c^** |  |  |  |  |  |  |
| Less intense (≤2 regimen) | - | - | - | 6 (15.0%) | 2 (9.1%) | 8 (12.9%) |
| Intense (3 or 4 regimen) | - | - | - | 34 (85.0%) | 20 (90.9%) | 54 (87.1%) |
| **Median days since transplant (IQR)^c^** |  |  |  | 1,067 (665-2,999) | 643 (287-2808) | 859 (462-2,881) |
| **SARS-CoV-2 specific monoclonal antibodies received within the last 6 months** | 0 | 0 | 0 | 3 (7.5%) | 1 (4.5%) | 4 (6.5%) |

^a^Only considering participants from the Swiss HIV Cohort Study

^b^Suppressed HIV viral load defined as <50 copies/ml

^c^Only considering participants from the Swiss Transplant Cohort Study

Abbreviations: IQR=Interquartile range; SHCS=Swiss HIV Cohort Study; STCT= Swiss Transplant Cohort Study

**Table S3: Antibody response before and after vaccination with bivalent mRNA SARS-CoV-2 vaccines in participants with different levels of immunosuppression, including only blood samples that were provided within the specified time window**

|  | **People living with HIV** | | | **Solid organ transplant recipients** | | |
| --- | --- | --- | --- | --- | --- | --- |
|  | **CD4 <350 cells/µL** | **CD4 ≥350 cells/µL** | **All** | **Kidney** | **Lung** | **All** |
| **Baseline** |  |  |  |  |  |  |
| Antibody response (cut-off >1642 units/ml) | 75% (56-94%) 15/20 | 89% (82-95%) 79/89 | 86% (80-93%) 94/109 | 67% (36-97%) 6/9 | 43% (26-59%) 15/35 | 48% (33-62%) 21/44 |
| Antibody response (cut-off >100 units/ml) | 90% (77-100%) 18/20 | 100% (NA) 89/89 | 98% (96-100%) 107/109 | 100% (NA) 9/9 | 83% (70-95%) 29/35 | 86% (76-97%) 38/44 |
| Antibody response (cut-off >0.8 units/ml) | 100% (NA) 20/20 | 100% (NA) 89/89 | 100% (NA) 109/109 | 100% (NA) 9/9 | 91% (82-100%) 32/35 | 93% (86-100%) 41/44 |
| Geometric mean concentrations (IQR)^a^ | 4,398 (1,406-13,764) n=18 | 8,992 (6,877-11,757) n=86 | 7,945 (5,941-10,625) n=104 | 21,795 (NA) n=1 | 875 (338-2,262) n=21 | 1,012 (390-2,626) n=22 |
| **4 Week follow-up** |  |  |  |  |  |  |
| Antibody response (cut-off >1642 units/ml) | 84% (68-100%) 16/19 | 100% (NA) 80/80 | 97% (94-100%) 96/99 | 92% (76-100%) 11/12 | 68% (50-86%) 17/25 | 76% (62-90%) 28/37 |
| Antibody response (cut-off >100 units/ml) | 95% (85-100%) 18/19 | 100% (NA) 80/80 | 99% (97-100%) 98/99 | 92% (76-100%) 11/12 | 92% (81-100%) 23/25 | 95% (87-100%) 35/37 |
| Antibody response (cut-off >0.8 units/ml) | 100% (NA) 19/19 | 100% (NA) 80/80 | 100% (NA) 99/99 | 100% (NA) 12/12 | 96% (88-100%) 24/25 | 97% (92-100%) 36/37 |
| Geometric mean concentrations (IQR)^a^ | 32,010 (11,762- 87,111) n=17 | 52,855 (43,972- 63,533) n=76 | 48,225 (38,392-60,577) n=93 | 36,347 (2,676-49,3701) n=4 | 3,412 (971-11,997) n=14 | 5,773 (1,873-17,794) n=18 |
| **8 Week follow-up** |  |  |  |  |  |  |
| Antibody response (cut-off >1642 units/ml) | 94% (82-100%) 15/16 | 100% (NA) 75/75 | 99% (97-100%) 90/91 | 95% (85-100%) 18/19 | 65% (48-81%) 20/31 | 76% (64-88%) 38/50 |
| Antibody response (cut-off >100 units/ml) | 100% (NA) 16/16 | 100% (NA) 75/75 | 100% (NA) 91/91 | 100% (NA) 19/19 | 94% (85-100%) 29/31 | 96% (91-100%) 48/50 |
| Antibody response (cut-off >0.8 units/ml) | 100% (NA) 16/16 | 100% (NA) 75/75 | 100% (NA) 91/91 | 100% (NA) 19/19 | 94% (85-100%) 29/31 | 96% (91-100%) 48/50 |
| Geometric mean concentrations (IQR)^a^ | 26,661 (13,656- 52,051) n=14 | 41,209 (33,616- 50,516) n=71 | 38,357 (31,436-46,801) n=85 | 31,287 (8,511-115,020) n=8 | 6,914 (3,201- 14,935) n=18 | 11,002 (5,587-21,666) n=26 |
| **6 months follow-up** |  |  |  |  |  |  |
| Antibody response (cut-off ≥1642 units/ml) | 82% (62-100%) 13/16 | 99% (96-100%) 70/71 | 95% (91-100%) 83/87 | 95% (85-100%) 19/20 | 67% (47-87%) 14/21 | 80% (68-93%) 33/41 |
| Antibody response (cut-off ≥100 units/ml) | 100% (NA) 16/16 | 100% (NA) 71/71 | 100% (NA) 87/87 | 100% (NA) 20/20 | 86% (71-100%) 18/21 | 93% (85- 100%) 38/41 |
| Antibody response (cut-off ≥0.8 units/ml) | 100% (NA) 16/16 | 100% (NA) 71/71 | 100% (NA) 87/87 | 100% (NA) 20/20 | 90% (78-100%) 19/21 | 95% (89-100%) 39/41 |
| Geometric mean concentrations (IQR)^a^ | 11,861 (5,283-26,625) n=14 | 16,398 (13,129-20,483) n=67 | 15,505 (12,403-19,385) n=81 | 15,831 (5,440-46,068) n=9 | 4,410 (1,560-12,461) n=8 | 8,675 (4,138-18,189) n=17 |

^a^Excluding study samples from the University Hospital Basel center as the maximum antibody concentration that was measured in the laboratory was 2500 units/mL.

Abbreviations: IQR=Interquartile range; NA=Not applicable

**Table S4: Antibody response before and after vaccination with bivalent mRNA SARS-CoV-2 vaccines in participants from the Swiss HIV Cohort Study**

|  | **mRNA-1273.214 by Moderna** | | | **BA.1–adapted BNT162b2 by Pfizer-BioNTech** | | |
| --- | --- | --- | --- | --- | --- | --- |
|  | **CD4 <350 cells/µL (n=6)** | **CD4 ≥350 cells/µL (n=55)** | **All (n=61)** | **CD4 <350 cells/µL (n=14)** | **CD4 ≥350 cells/µL (n=37)** | **All (n=51)** |
| **Baseline** |  |  |  |  |  |  |
| Antibody response (cut-off >1642 units/ml) | 67% (29-100%) 4/6 | 89% (81- 97%) 48/54 | 87% (78-95%) 52/60 | 79% (57-100%) 11/14 | 89% (79-99%) 33/37 | 86% (77- 96%) 44/51 |
| Antibody response (cut-off >100 units/ml) | 67% (29-100%) 4/6 | 100% (NA) 54/54 | 97% (92-100%) 58/60 | 100% (NA) 14/14 | 100% (NA) 37/37 | 100% (NA) 51/51 |
| Antibody response (cut-off >0.8 units/ml) | 100% (NA) 6/6 | 100% (NA) 54/54 | 100% (NA) 60/60 | 100% (NA) 14/14 | 100% (NA) 37/37 | 100% (NA) 51/51 |
| Geometric mean concentrations (IQR)^a^ | 1,316 (14-127,398) n=5 | 9,104 (6,299- 13,158) n=51 | 7,660 (4,872-12,043) n=56 | 6,996 (2,917-16,775) n=13 | 8,837 (5,966-13,089) n=36 | 7,955 (5,965-10,608) n=105 |
| **4 Week follow-up** |  |  |  |  |  |  |
| Antibody response (cut-off >1642 units/ml) | 67% (29-100%) 4/6 | 100% (NA) 51/51 | 96% (92-100%) 55/57 | 93% (79-100%) 13/14 | 100% (NA) 35/35 | 98% (94-100%) 48/49 |
| Antibody response (cut-off >100 units/ml) | 100% (NA) 6/6 | 100% (NA) 51/51 | 100% (NA) 57/57 | 93% (79-100%) 13/14 | 100% (NA) 35/35 | 98% (94-100%) 48/49 |
| Antibody response (cut-off >0.8 units/ml) | 100% (NA) 6/6 | 100% (NA) 51/51 | 100% (NA) 57/57 | 100% (NA) 14/14 | 100% (NA) 35/35 | 100% (NA) 49/49 |
| Geometric mean concentrations (IQR)^a^ | 30,453 (3,240-286,239) n=5 | 55,179 (42,379-71,846) n=48 | 52,170 (39,450-68,992) n=53 | 33,100 (9,928-110,347) n=13 | 51,126 (39,546-66,096) n=34 | 48,837 (39,209-60,830) n=100 |
| **8 Week follow-up** |  |  |  |  |  |  |
| Antibody response (cut-off >1642 units/ml) | 80% (45-100%) 4/5 | 98% (94-100%) 48/49 | 96% (91-100%) 52/54 | 100% (NA) 13/13 | 100% (NA) 34/34 | 100% (NA) 47/47 |
| Antibody response (cut-off >100 units/ml) | 100% (NA) 5/5 | 100% (NA) 49/49 | 100% (NA) 54/54 | 100% (NA) 13/13 | 100% (NA) 34/34 | 100% (NA) 47/47 |
| Antibody response (cut-off >0.8 units/ml) | 100% (NA) 5/5 | 100% (NA) 49/49 | 100% (NA) 54/54 | 100% (NA) 13/13 | 100% (NA) 34/34 | 100% (NA) 47/47 |
| Geometric mean concentrations (IQR)^a^ | 31,618 (5,244-190,642) n=4 | 34,905 (25,381-48,004) n=46 | 34,630 (25,579-46,883) n=50 | 26,699 (13,069-54,543) n=12 | 39,051 (28,916-52,739) n=33 | 34,939 (28,505-42,826) n=95 |
| **6 months follow-up** |  |  |  |  |  |  |
| Antibody response (cut-off ≥1642 units/ml) | 80% (45-100%) 4/5 | 98% (94-100%) 46/47 | 96% (91-100%) 50/52 | 83% (62-100%) 10/12 | 100% (NA) 36/36 | 96% (90-100%) 46/48 |
| Antibody response (cut-off ≥100 units/ml) | 100% (NA) 5/5 | 100% (NA) 47/47 | 100% (NA) 52/52 | 100% (NA) 12/12 | 100% (NA) 36/36 | 100% (NA) 48/48 |
| Antibody response (cut-off ≥0.8 units/ml) | 100% (NA) 5/5 | 100% (NA) 47/47 | 100% (NA) 52/52 | 100% (NA) 12/12 | 100% (NA) 36/36 | 100% (NA) 48/48 |
| Geometric mean concentrations (IQR)^a^ | 20,527 (4,282-98,409) n=4 | 17,088 (12,821-22,776) n=44 | 17,351 (13,211-22,790) n=48 | 10,353 (3,851-27,833) n=11 | 19,429 (13,801-27,351) n=35 | 17,036 (13,774-21,071) n=94 |

^a^Excluding study samples from the University Hospital Basel center as the maximum antibodies that was measured in the lab was 2500 units/mL

Abbreviations: IQR=Interquartile range; NA=Not applicable

**Table S5: Antibody response before and after vaccination with bivalent mRNA SARS-CoV-2 vaccines in participants from the Swiss Transplant Cohort Study**

|  | **mRNA-1273.214 by Moderna** | | | **BA.1–adapted BNT162b2 by Pfizer-BioNTech** | | |
| --- | --- | --- | --- | --- | --- | --- |
|  | **Kidney (n=11)** | **Lung (n=29)** | **All (n=40)** | **Kidney (n=11)** | **Lung (n=11)** | **All (n=22)** |
| **Baseline** |  |  |  |  |  |  |
| Antibody response (cut-off >1642 units/ml) | 73% (46-99%) 8/11 | 41% (23-59%) 12/29 | 50% (35-65%) 20/40 | 82% (59-100%) 9/11 | 67% (36-97%) 6/9 | 75% (56-94%) 15/20 |
| Antibody response (cut-off >100 units/ml) | 100% (NA) 11/11 | 83% (69-97%) 24/29 | 88% (77-98%) 35/40 | 100% (NA) 11/11 | 89% (68-100%) 8/9 | 95% (85-100%) 19/20 |
| Antibody response (cut-off >0.8 units/ml) | 100% (NA) 11/11 | 90% (79-100%) 26/29 | 93% (84-100%) 37/40 | 100% (NA) 11/11 | 100% (NA) 9/9 | 100% (NA) 20/20 |
| Geometric mean concentrations (IQR)^a^ | 5,997 (197-182,140) n=2 | 1,351 (497-3,674) n=15 | 1,610 (650-3,986) n=17 | 4,900 (1,259-19,072) n=8 | 1,044 (123-8,843) n=9 | 2,161 (637-7,338) n=17 |
| **4 Week follow-up** |  |  |  |  |  |  |
| Antibody response (cut-off >1642 units/ml) | 90% (71-100%) 9/10 | 61% (43-79%) 17/28 | 68% (54-83%) 26/38 | 100% (NA) 11/11 | 75% (45-100%) 6/8 | 89% (76-100%) 17/19 |
| Antibody response (cut-off >100 units/ml) | 100% (NA) 10/10 | 82% (68-96%) 23/28 | 87% (76-98%) 33/38 | 100% (NA) 11/11 | 88% (65-100%) 7/8 | 95% (85-100%) 18/19 |
| Antibody response (cut-off >0.8 units/ml) | 100% (NA) 10/10 | 89% (78-100%) 25/28 | 92% (84-100%) 35/38 | 100% (NA) 11/11 | 100% (NA) 8/8 | 100% (NA) 19/19 |
| Geometric mean concentrations (IQR)^a^ | 55,108 (242-12,542,979) n=2 | 4,033 (1,060-15,346) n=15 | 5,485 (1,578-19,069) n=17 | 19,452 (4,981-75,975) n=8 | 1,908 (298-12,229) n=8 | 6,092 (1,855-20,012) n=16 |
| **8 Week follow-up** |  |  |  |  |  |  |
| Antibody response (cut-off >1642 units/ml) | 91% (74-100%) 10/11 | 57% (39-75%) 16/28 | 67% (52-81%) 26/39 | 100% (NA) 11/11 | 75% (45-100%) 6/8 | 89% (76-100%) 17/19 |
| Antibody response (cut-off >100 units/ml) | 100% (NA) 11/11 | 86% (73-99%) 24/28 | 90% (80-99%) 35/39 | 100% (NA) 11/11 | 100% (NA) 8/8 | 100% (NA) 19/19 |
| Antibody response (cut-off >0.8 units/ml) | 100% (NA) 11/11 | 89% (78-100%) 25/28 | 92% (84-100%) 36/39 | 100% (NA) 11/11 | 100% (NA) 8/8 | 100% (NA) 19/19 |
| Geometric mean concentrations (IQR)^a^ | 36,961 (111- 12,292,699) n=2 | 4,306 (976-18,993) n=14 | 5,633 (1,480-21,444) n=16 | 20,942 (4,897-89,560) n=8 | 4,062 (1,462-11,289) n=8 | 9,223 (3,766-22,586) n=16 |
| **6 months follow-up** |  |  |  |  |  |  |
| Antibody response (cut-off ≥1642 units/ml) | 90% (71-100%) 9/10 | 65% (47-84%) 17/26 | 72% (58-87%) 26/36 | 100% (NA) 11/11 | 89% (68-100%) 8/9 | 95% (85-100%) 19/20 |
| Antibody response (cut-off ≥100 units/ml) | 100% (NA) 10/10 | 88% (76-100%) 23/26 | 92% (83-100%) 33/36 | 100% (NA) 11/11 | 100% (NA) 9/9 | 100% (NA) 20/20 |
| Antibody response (cut-off ≥0.8 units/ml) | 100% (NA) 10/10 | 92% (82-100%) 24/26 | 94% (87-100%) 34/36 | 100% (NA) 11/11 | 100% (NA) 9/9 | 100% (NA) 20/20 |
| Geometric mean concentrations (IQR)^a^ | 12,700 (598-269,886) n=2 | 6,174 (2,654-14,363) n=13 | 6,797 (3,270-14,130) n=15 | 12,950 (3,254-51,535) n=8 | 4,837 (1,948-12,009) n=9 | 7,689 (3,617-16,342) n=17 |

^a^Excluding study samples from the University Hospital Basel center as the maximum antibodies that was measured in the lab was 2500 units/mL

Abbreviations: IQR=Interquartile range; NA=Not applicable

**Table S6: Antibody response before and after vaccination with bivalent mRNA SARS-CoV-2 vaccines, stratified by participants who had a positive antibody test to the nucleocapsid protein or a SARS-CoV-2 vaccine in the past 6 month**

|  | **People living with HIV** | | | **Solid organ transplant recipients** | | |
| --- | --- | --- | --- | --- | --- | --- |
|  | **Antibody test to the nucleocapsid protein positive or SARS-CoV-2 vaccine in past 6 months** | | | **Antibody test to the nucleocapsid protein positive or SARS-CoV-2 vaccine in past 6 months** | | |
|  | **No (n=37)** | **Yes (n=75)** | **All (n=112)** | **No (n=35)** | **Yes (n=27)** | **All (n=62)** |
| **Baseline** |  |  |  |  |  |  |
| Antibody response (cut-off >1642 units/ml) | 67% (51-82%) 24/36 | 96% (92-100%) 72/75 | 86% (80-93%) 96/111 | 39% (23-56%) 13/33 | 81% (67-96%) 22/27 | 58% (46-71%) 35/60 |
| Antibody response (cut-off >100 units/ml) | 97% (92-100%) 35/36 | 99% (96-100%) 74/75 | 98% (96-100%) 109/111 | 82% (69-95%) 27/33 | 100% (NA) 27/27 | 90% (82-98%) 54/60 |
| Antibody response (cut-off >0.8 units/ml) | 100% (NA) 36/36 | 100% (NA) 75/75 | 100% (NA) 111/111 | 91% (81-100%) 30/33 | 100% (NA) 27/27 | 95% (89-100%) 57/60 |
| Geometric mean concentrations (IQR)^a^ | 2,976 (1,716-5,161) n=36 | 11,625 (8,900-15,185) n=75 | 7,472 (5,667-9,852) n=111 | 443 (156-1,253) n=33 | 3,022 (1,915-4,769) n=27 | 1051 (553-1,998) n=60 |
| **4 Week follow-up** |  |  |  |  |  |  |
| Antibody response (cut-off >1642 units/ml) | 91% (81-100%) 29/32 | 100% (NA) 74/74 | 97% (94-100%) 103/106 | 70% (54-85%) 23/33 | 83% (68-98%) 20/24 | 75% (64-87%) 43/57 |
| Antibody response (cut-off >100 units/ml) | 97% (91-100%) 31/32 | 100% (NA) 74/74 | 99% (97-100%) 105/106 | 82% (69-95%) 27/33 | 100% (NA%) 24/24 | 89% (81-97%) 51/57 |
| Antibody response (cut-off >0.8 units/ml) | 100% (NA) 32/32 | 100% (NA) 74/74 | 100% (NA) 106/106 | 91% (81-100%) 30/33 | 100% (NA%) 24/24 | 95% (89-100%) 54/57 |
| Geometric mean concentrations (IQR)^a^ | 28,058 (14,289-55,095) n=32 | 47,028 (36,956-59,846) n=74 | 40,239 (30,980-52,264) n=106 | 1,040 (343-3,151) n=33 | 7,550 (3,751-15,199) n=24 | 2,396 (1,148-5,000) n=57 |
| **8 Week follow-up** |  |  |  |  |  |  |
| Antibody response (cut-off >1642 units/ml) | 94% (86-100%) 31/33 | 100% (NA) 68/68 | 98% (95-100%) 99/101 | 68% (52-83%) 23/34 | 83% (68-98%) 20/24 | 74% (63-85%) 43/58 |
| Antibody response (cut-off >100 units/ml) | 100% (NA) 33/33 | 100% (NA) 68/68 | 100% (NA) 101/101 | 88% (77-99%) 30/34 | 100% (NA) 24/24 | 93% (87-100%) 54/58 |
| Antibody response (cut-off >0.8 units/ml) | 100% (NA) 33/33 | 100% (NA) 68/68 | 100% (NA) 101/101 | 91% (82-100%) 31/34 | 100% (NA) 24/24 | 95% (89-100%) 55/58 |
| Geometric mean concentrations (IQR)^a^ | 23,379 (13,444-40,657) n=33 | 32,206 (24,871-41,704) n=68 | 29,006 (22,657-37,134) n=101 | 1,253 (435-3,608) n=34 | 7,417 (3,596-15,295) n=24 | 2,615 (1,286-5,318) n=58 |
| **6 months follow-up** |  |  |  |  |  |  |
| Antibody response (cut-off ≥1642 units/ml) | 91% (81-100%) 30/33 | 99% (96-100%) 66/67 | 96% (92-100%) 96/100 | 71% (55-87%) 22/31 | 92% (82-100%) 23/25 | 80% (70-91%) 45/56 |
| Antibody response (cut-off ≥100 units/ml) | 100% (NA) 33/33 | 100% (NA) 67/67 | 100% (NA) 100/100 | 90% (80-100%) 28/31 | 100% (NA) 25/25 | 95% (89-100%) 53/56 |
| Antibody response (cut-off ≥0.8 units/ml) | 100% (NA) 33/33 | 100% (NA) 67/67 | 100% (NA) 100/100 | 94% (85-100%) 29/31 | 100% (NA) 25/25 | 96% (92-100%) 54/56 |
| Geometric mean concentrations (IQR)^a^ | 21,603 (10,327-32,878) n=33 | 4,162 (153-8,170) n=67 | 9,917 (5,16-14,674) n=100 | 1,183 (428-3,267) n=31 | 6,689 (3,873-11,552) n=25 | 2,563 (1,352-4,860) n=56 |

^a^Excluding study samples from the University Hospital Basel center as the maximum antibodies that was measured in the lab was 2500 units/mL

Abbreviations: IQR=Interquartile range; NA=Not applicable

**Table S7: Characteristics of the 17 participants with an antibody response below 1642 units/ml eight weeks after receiving a bivalent SARS-CoV-2 vaccine**

^a^Only considering participants from the Swiss HIV Cohort Study

^b^Only considering participants from the Swiss Transplant Cohort Study

Abbreviations: SHCS = Swiss HIV Cohort Study; STCT = Swiss Transplant Cohort Study; NE = not evaluable; R=Regimens

**Table S8: Baseline characteristics before receiving a bivalent mRNA SARS-CoV-2 vaccine for the participants included in the T-cell sub-study**

| **Characteristics** | **mRNA-1273.214 by Moderna** (n=54) | **BA.1–adapted BNT162b2 by Pfizer-BioNTech** (n=27) | **Total**  (n=81) |
| --- | --- | --- | --- |
| **Median age (IQR)** | 55 (45-63) | 61 (51-66) | 57 (46-64) |
| **Sex** |  |  |  |
| Male | 36/54 (66.7%) | 22/27 (81.5%) | 58 (71.6%) |
| Female | 18/54 (33.3%) | 5/27 (18.5%) | 23 (28.4%) |
| **Cohort** |  |  |  |
| SHCS | 17/54 (31.5%) | 18/27 (66.7%) | 35 (43.2%) |
| STCS | 37/54 (68.5%) | 9/27 (33.3%) | 46 (56.8%) |
| **Antibody test to the nucleocapsid protein** |  |  |  |
| Non-reactive | 27/54 (50.0%) | 10/27 (37.0%) | 37 (45.7%) |
| Reactive | 27/54 (50.0%) | 16/27 (59.3%) | 43 (53.1%) |
| Missing | 0/54 | 1/27 (3.7%) | 1 (1.2%) |
| **Previous SARS-CoV-2 vaccine in the past 6 months** | 2/54 (3.7%) | 3/27 (11.1%) | 5/81 (6.2%) |
| **History of cardiovascular disease or metabolic syndrome^a^** | 38/54 (70.4%) | 16/27 (59.3%) | 54 (66.7%) |
| **Number of previously received SARS-CoV-2 vaccines** |  |  |  |
| 2 | 2/54 (3.7%) | 1/27 (3.7%) | 3 (3.7%) |
| 3 | 43/54 (79.6%) | 20/27 (74.1%) | 63 (77.8%) |
| 4 | 8/54 (14.8%) | 6/27 (22.2%) | 14 (17.3%) |
| 5 | 1/54 (1.9%) | 0/27 (0.0%) | 1 (1.2%) |
| **Seasonal flu vaccine (2022/2023) received** | 28/54 (51.9%) | 12/27 (44.4%) | 40 (49.4%) |
| **CD4 cell count (cells/µL)^a^** |  |  |  |
| <350 | 3/17 (17.6%) | 8/18 (44.4%) | 11/35 (31.4%) |
| ≥350 | 14/17 (82.4%) | 10/18 (55.6%) | 24/35 (68.6%) |
| **Suppressed HIV viral load^ab^** | 17/17 (100.0%) | 18/18 (100.0%) | 35/35 (100.0%) |
| **Transplanted organ^c^** |  |  |  |
| Kidney transplant | 9/37 (24.3%) | 2/9 (22.2%) | 11/46 (23.9%) |
| Lung transplant | 28/37 (75.7%) | 7/9 (77.8%) | 35/46 (76.1%) |
| **Current Immunosuppressive therapy^c^** |  |  |  |
| Less intense (≤2 regimen)^d^ | 6/37 (16.2%) | 0/9 (0.0%) | 6/46 (13.0%) |
| Intense (3 or 4 regimen)^d^ | 31/37 (83.8%) | 9/9 (100.0%) | 40/46 (87.0%) |
| **Median days since transplant (IQR)^c^** | 1,300 (736-3,012) | 875 (355-2,881) | 1,219 (616-3,008) |
| **SARS-CoV-2 specific monoclonal antibodies received within the last 6 months** | 3/54 (5.6%) | 1/27 (3.7%) | 4/81 (4.9%) |

^a^Only considering participants from the Swiss HIV Cohort Study

^b^Suppressed HIV viral load defined as <50 copies/ml

^c^Only considering participants from the Swiss Transplant Cohort Study

^d^Intense treatment defined as triple or quadruple immunosuppressive regimen vs. less intense immunosuppressive therapy defined as dual immunosuppressive regimen

Abbreviations: IQR=Interquartile range; SHCS=Swiss HIV Cohort Study; STCT= Swiss Transplant Cohort Study

**Table S9: T-cell response before and after vaccination with bivalent mRNA SARS-CoV-2 vaccines in a sub-sample of participants with different levels of immunosuppression, including only patients that provided a blood sample within the specified time window**

|  | **People living with HIV** | | | **Solid organ transplant recipients** | | |
| --- | --- | --- | --- | --- | --- | --- |
|  | **CD4 <350** | **CD4 ≥350** | **All** | **Kidney** | **Lung** | **All** |
| **Baseline** |  |  |  |  |  |  |
| Positive | 90%; 9/10 | 100%; 17/17 | 96%; 26/27 | 63%; 5/8 | 29%; 9/31 | 36%; 14/39 |
| Borderline | 10%; 1/10 | 0%; 0/17 | 4%; 1/27 | 0%; 0/8 | 3%; 1/31 | 3%; 1/39 |
| Negative | 0%; 0/10 | 0%; 0/17 | 0%; 0/27 | 38%; 3/8 | 19%; 6/31 | 23%; 9/39 |
| Not evaluable | 0%; 0/10 | 0%; 0/17 | 0%; 0/27 | 0%; 0/8 | 48%; 15/31 | 38%; 15/39 |
| Geometric mean concentration (IQR)^a^ | 1,193 (448-3,179) n=10 | 2,202 (1,234-3,931) n=17 | 1755 (1077-2859) n=27 | 197 (62-624) n=8 | 37 (2-653) n=16 | 65 (10-430) n=24 |
| **4 Week follow-up** |  |  |  |  |  |  |
| Positive | 70% (42-98%) 7/10 | 95% (87-100%) 21/22 | 88% (76-99%) 28/32 | 50% (15-85%) 4/8 | 38% (18-57%) 9/24 | 41% (24-58%) 13/32 |
| Borderline | 10% (0-29%) 1/10 | 0% (NA) 0/22 | 3% (0-9%) 1/32 | 13% (0-35%) 1/8 | 4% (0-12%) 1/24 | 6% (0-15%) 2/32 |
| Negative | 0% (NA) 0/10 | 0% (NA) 0/22 | 0% (NA) 0/32 | 25% (0-55%) 2/8 | 17% (2-32%) 4/24 | 19% (5-32%) 6/32 |
| Not evaluable | 20% (0-45%) 2/10 | 5% (0-13%) 1/22 | 9% (0-19%) 3/32 | 13% (0-35%) 1/8 | 42% (22-61%) 10/24 | 34% (18-51%) 11/32 |
| Geometric mean concentration (IQR)^a^ | 1,326 (546-3,217) n=8 | 3,933 (2,427-6,371) n=20 | 2,882 (1,856-4,477) n=28 | 174 (57-531) n=7 | 73 (2-2,420) n=14 | 97 (10-928) n=21 |
| **6 Months follow-up** |  |  |  |  |  |  |
| Positive | 75% (45-100%) 6/8 | 94% (83-100%) 16/17 | 88% (75-100%) 22/25 | 50% (19-81%) 5/10 | 44% (21-67%) 8/18 | 46% (28-65%) 13/28 |
| Borderline | 25% (0-55%) 2/8 | 0% (NA) 0/17 | 8% (0-19%) 2/25 | 30% (2-58%) 3/10 | 11% (0-26%) 2/18 | 18% (4-32%) 5/28 |
| Negative | 0% (NA) 0/8 | 6% (0.17%) 1/17 | 4% (0-12%) 1/25 | 10% (0-29%) 1/10 | 6% (0-16%) 1/18 | 7% (0-17%) 2/28 |
| Not evaluable | 0% (NA) 0/8 | 0% (NA) 0/17 | 0% (0-0%) 0/25 | 10% (0-29%) 1/10 | 39% (16-61%) 7/18 | 29% (12-45%) 8/28 |
| Geometric mean concentration (IQR)^a^ | 449 (174-1,158) n=7 | 1,666 (1,036-2,678) n=14 | 1,076 (659-1,755) n=21 | 368 (118; 1152) n=9 | 115 (5-2,725) n=11 | 97 (11-836) n=20 |

^a^Excluding not evaluable patients

Abbreviations: IQR=Interquartile range; NA=Not applicable

**Table S10: T-cell response before and after vaccination with bivalent mRNA SARS-CoV-2 vaccines in a sub-sample of participants with different levels of immunosuppression, stratified by participants who had a positive antibody test to the nucleocapsid protein or a SARS-CoV-2 vaccine in the past 6 month**

|  | **People living with HIV** | | | **Solid organ transplant recipients** | | |
| --- | --- | --- | --- | --- | --- | --- |
|  | **Antibody test to the nucleocapsid protein positive or SARS-CoV-2 vaccine in past 6 months** | | | **Antibody test to the nucleocapsid protein positive or SARS-CoV-2 vaccine in past 6 months** | | |
|  | **No** | **Yes** | **All** | **No** | **Yes** | **All** |
| **Baseline** |  |  |  |  |  |  |
| Positive | 89% (68-100%) 8/9 | 100% (NA) 18/18 | 96% (89-100%) 26/27 | 30% (12-47%) 8/27 | 44% (21-67%) 8/18 | 36% (22-50%) 16/45 |
| Borderline | 11% (0-32%) 1/9 | 0% (NA) 0/18 | 3.7% (0; 10.83%) 1/27 | 7% (0-17%) 2/27 | 0% (NA) 0/18 | 4% (0-10%) 2/45 |
| Negative | 0% (NA) 0/9 | 0% (NA) 0/18 | 0% (0; 0%) 0/27 | 19% (3-33%) 5/27 | 22% (3-41%) 4/18 | 20% (8-32%) 9/45 |
| Not evaluable | 0% (NA) 0/9 | 0% (NA) 0/18 | 0% (0; 0%) 0/27 | 44% (26-63%) 12/27 | 33% (12-55%) 6/18 | 40% (26-54%) 18/45 |
| Geometric mean concentration (IQR)^a^ | 935 (415; 2107) n=9 | 2404 (1311; 4409) n=18 | 1755 (1077; 2859) n=27 | 85 (9-795) n=15 | 85 (4-1,767) n=12 | 85 (16-464) n=27 |
| **4 Week follow-up** |  |  |  |  |  |  |
| Positive | 80% (55-100%) 8/10 | 92% (81-100%) 23/25 | 89% (78-99%) 31/35 | 44% (26-63%) 12/27 | 31% (9-54%) 5/16 | 40% (25-54%) 17/43 |
| Borderline | 0% (NA) 0/10 | 4% (0-12%) 1/25 | 3% (0-8%) 1/35 | 4% (0-11%) 1/27 | 6% (0-18%) 1/16 | 5% (0-11%) 2/43 |
| Negative | 0% (NA) 0/10 | 0% (NA) 0/25 | 0% (NA) 0/35 | 19% (4-33%) 5/27 | 25% (4-46%) 4/16 | 21% (9-33%) 9/43 |
| Not evaluable | 20% (0-45%) 2/10 | 4% (0-12%) 1/25 | 9% (0-18%) 3/35 | 33% (16-51%) 9/27 | 38% (14-61%) 6/16 | 35% (21-49%) 15/43 |
| Geometric mean concentration (IQR)^a^ | 2,990 (1,421-6,292) n=8 | 3,189 (1,908-5,332) n=24 | 3,138 (2,088-4,718) n=32 | 77 (6-1,015) n=18 | 24 (0-3,165) n=10 | 51 (5-467) n=28 |
| **6 Months follow-up** |  |  |  |  |  |  |
| Positive | 86% (60-100%) 6/7 | 94% (82-100%) 15/16 | 91% (80-100%) 21/23 | 52% (31.76; 72.59%) 12/23 | 53.33% (28.09; 78.58%) 8/15 | 52.63% (36.76; 68.51%) 20/38 |
| Borderline | 14% (0-40%) 1/7 | 6% (0-18%) 1/16 | 9% (0-20%) 2/23 | 17.39% (1.9; 32.88%) 4/23 | 13.33% (0; 30.54%) 2/15 | 15.79% (4.2; 27.38%) 6/38 |
| Negative | 0% (NA) 0/7 | 0% (NA) 0/16 | 0% (NA) 0/23 | 4.35% (0; 12.68%) 1/23 | 13.33% (0; 30.54%) 2/15 | 7.89% (0; 16.47%) 3/38 |
| Not evaluable | 0% (NA) 0/7 | 0% (NA) 0/16 | 0% (NA) 0/23 | 26.09% (8.14; 44.03%) 6/23 | 20% (0; 40.24%) 3/15 | 23.68% (10.17; 37.2%) 9/38 |
| Geometric mean concentration (IQR)^a^ | 893 (322-2,478) n=7 | 1,392 (731-2,649) n=16 | 1,216 (733-2,017) n=23 | 472 (250; 892) n=17 | 82 (5; 1323) n=12 | 229 (72; 724) n=29 |

^a^Excluding not evaluable patients

Abbreviations: IQR=Interquartile range; NA=Not applicable

**Table S11: Characteristics of the eight participants with a confirmed SARS-CoV-2 infection**

| **Characteristics** | **Participants** | | | | | | | |
| --- | --- | --- | --- | --- | --- | --- | --- | --- |
|  | **1** | **2** | **3** | **4** | **5** | **6** | **7** | **8** |
| **Cohort** | SHCS | STCS | STCS | STCS | STCS | STCS | STCS | STCS |
| **Age (years)** | 59 | 36 | 48 | 58 | 62 | 68 | 61 | 59 |
| **Sex** | Male | Male | Male | Female | Male | Female | Male | Male |
| **CD4/CD8 (cells/µL)** | 272/431 | - | - | - | - | - | - | - |
| **CD4:CD8 ratio^a^** | 0.63 | - | - | - | - | - | - | - |
| **Suppressed HIV viral load (<50 copies/ml)^a^** | Yes | - | - | - | - | - | - | - |
| **Transplanted organ^b^** | - | Lung | Lung | Lung | Lung | Lung | Kidney | Lung |
| **Immunosuppressive therapy^b^** | - | 3 or 4 regimens | 3 or 4 regimens | 3 or 4 regimens | ≤2 regimens | 3 or 4 regimens | 3 or 4 regimens | 3 or 4 regimens |
| **Number of previously received SARS-CoV-2 vaccines** | 3 | 3 | 2 | 3 | 3 | 3 | 3 | 3 |
| **Bivalent SARS-CoV-2 product** | Pfizer-BioNTech | Moderna | Moderna | Moderna | Moderna | Moderna | Moderna | Pfizer-BioNTech |
| **Study visit when SARS-CoV-2 infection was reported** | 6 months | 6 months | 6 months | 6 months | 6 months | 6 months | 4 weeks | 4 weeks |
| **Flu vaccine (assessed at baseline)** | No | No | No | Yes | No | Yes | No | Yes |
| **Monoclonal treatment (assessed at baseline)** | No | No | No | No | No | No | No | No |
| **Anti-spike antibody concentrations at study visit before SARS-CoV-2 infection was diagnosed (U/mL)** | 70175 | 2500 | 30525 | 719 | 3665 | 19265 | 2500 | 2307 |
| **T-cell concentration at study visit before SARS-CoV-2 infection was diagnosed (mlU/mL)** | 3726^d^ | 1020^d^ | 973^d^ | 0^d^ | NE^d^ | NE^d^ | 235^c^ | NE^c^ |
| **T-cell concentration at study visit after SARS-CoV-2 infection was diagnosed (mlU/mL)** | 1250 | NE | 1904 | 0 | 573 | 572 | 64.8 | NE |
| **Monoclonal treatment after SARS-CoV-2 infection** | No | No | No | No | No | No | No | No |

^a^Only considering participants from the Swiss HIV Cohort Study

^b^Only considering participants from the Swiss Transplant Cohort Study

^c^Baseline values, infection was reported at 4w follow-up; vaccine was given in between

^d^Values from the 4 week study visit

Abbreviations: SHCS = Swiss HIV Cohort Study; STCT = Swiss Transplant Cohort Study; NE = not evaluable
